# Polyphenol-Microbiota Interactions in Atherosclerosis: The Role of Hydroxytyrosol and Tyrosol in Modulating Inflammation and Oxidative Stress

**DOI:** 10.3390/nu17233784

**Published:** 2025-12-02

**Authors:** Mojgan Morvaridzadeh, Mehdi Alami, Hicham Berrougui, Kaoutar Boumezough, Hawa Sidibé, Ikram Salih, Khalid Sadki, Abdelouahed Khalil

**Affiliations:** 1 Geriatrics Unit, Department of Medicine, Faculty of Medicine and Health Sciences, University of Sherbrooke, Sherbrooke, QC J1H 4N4, Canada; mojgan.morvaridzadeh@usherbrooke.ca (M.M.); mehdi.alami@usherbrooke.ca (M.A.); hicham.berrougui@usherbrooke.ca (H.B.); kaoutar.boumezough@usherbrooke.ca (K.B.); hawa.sidibe@usherbrooke.ca (H.S.); ikram.salih@usherbrooke.ca (I.S.); 2 Department of Biology, Polydisciplinary Faculty, University Sultan Moulay Slimane, Beni Mellal 23000, Morocco; 3 Research Laboratory in Oral Biology and Biotechnology, Faculty of Dental Medicine, Mohammed V University in Rabat, Rabat 10000, Morocco; k.sadki@um5r.ac.ma

**Keywords:** tyrosol, hydroxytyrosol, extra virgin olive oil, atherosclerosis, gut microbiota

## Abstract

Atherosclerosis is a chronic inflammatory cardiovascular disease that may result from the interaction between oxidative stress, immune dysregulation, and metabolic disorders. Recent studies indicate that the well-known phenolic compounds, hydroxytyrosol (HTyr) and tyrosol (Tyr) present in extra virgin olive oil, confer cardioprotection through various mechanisms of action that include antioxidant, anti-inflammatory, and metabolic regulatory properties. The gut microbiota modulates the structure, bioavailability, and bioactivity of these phenolic compounds, thereby influencing their therapeutic potential. This review explores the intricate interactions between Tyr, HTyr, and gut microbiota within the context of atherosclerosis prevention and management. We explore how gut microbial metabolism can magnify or alter the biological effects of the Tyr and HTyr, and how interindividual differences in microbiota composition may influence their efficacy. A deeper understanding of these mechanisms could support the development of precision nutrition strategies aimed at reducing the risk of atherosclerosis.

## 1. Introduction

Cardiovascular disease (CVD) remains the leading cause of mortality worldwide, accounting for approximately 32% of annual deaths, with atherosclerotic cardiovascular disease (ASCVD) as the primary contributor [1,2]. Atherosclerosis is a chronic inflammatory condition affecting medium and large arteries and underlies clinical events such as myocardial infarction, stroke, and peripheral artery disease [3]. It is characterized by lipid accumulation, endothelial dysfunction, oxidative stress, and immune activation, ultimately leading to plaque formation and, in advanced stages, thrombotic complications [4]. Major risk factors include hypercholesterolemia, hypertension, diabetes, infections, and smoking [5,6].

Dietary phenolics such as HTyr and Tyr have been investigated for their vascular and metabolic benefits [7,8]. However, findings from human intervention studies remain inconsistent, largely due to substantial variability in phenolic composition of foods, differences in absorption and metabolism, limitations in dietary assessment, population heterogeneity, and interindividual variation in gut microbiota composition [9,10].

The gut microbiota, consisting of over 100 trillion microbial cells, plays a fundamental role in host metabolism, immune function, and overall cardiovascular health [11]. Emerging evidence highlights a bidirectional relationship between the gut microbiota and dietary polyphenols, particularly in the modulation of inflammation and lipid metabolism; two central processes in atherosclerosis [12,13]. Dysbiosis has been increasingly associated with CVD development and atherosclerosis progression [14,15,16]. Importantly, gut microbiota composition may strongly influence the bioavailability, bioactivity, and ultimately the cardiometabolic efficacy of Tyr and HTyr [17].

Building on this premise, this review provides a detailed analysis of the mechanistic interplay between Tyr/HTyr and gut microbiota, with a particular emphasis on how microbial metabolism shapes the vascular effects of these phenolic alcohols. Clarifying these interactions may help explain inconsistent trial results and support microbiome-informed nutritional strategies.

Compared with previous reviews on olive oil polyphenols and cardiovascular health, this article offers a more targeted and updated synthesis on HTyr and Tyr, integrates gut microbiota-dependent metabolism and interindividual variability, and aligns mechanistic insights with available human clinical and dose–response evidence to identify translational gaps and opportunities for precision nutrition.

This narrative review is based on a literature search conducted in PubMed, Scopus, and the Cochrane Library for peer-reviewed articles of randomized clinical trials published in English from 2010 to October 2025. The search combined the following keywords: “hydroxytyrosol” OR “tyrosol” OR “oleuropein” OR “olive oil polyphenols” AND “gut microbiota” OR “microbiome” AND “atherosclerosis” OR “cardiovascular disease” OR “oxidative stress” OR “inflammation” OR “metabolism”. Additional studies were identified by screening reference lists of relevant articles and reviews. Experimental (in vitro and in vivo), mechanistic, and human interventional studies were included when assessing outcomes related to oxidative stress, inflammation, endothelial function, lipid or glucose metabolism, or gut microbiota composition or functionality. Although this is a narrative review, a formal risk-of-bias assessment (RoB2) was conducted for the included randomized clinical trials to support a transparent appraisal of study quality. No meta-analysis was performed; instead, the evidence is synthesized qualitatively, with an emphasis on mechanistic coherence and translational relevance.

## 2. Metabolism and Bioavailability of Hydroxytyrosol and Tyrosol

Polyphenols are a diverse group of bioactive compounds widely distributed in fruits, vegetables, cereals, tea, coffee, and wine [18]. Beyond their health-related properties, they also influence sensory characteristics such as bitterness and contribute to oxidative stability in food products [19]. Phenolic compounds are commonly classified into two major categories: flavonoids (including anthocyanins, flavanols, flavanones, flavonols, and isoflavones) and non-flavonoids (such as phenolic acids, xanthones, stilbenes, lignans, and tannins) [20].

### 2.1. Chemical Structure

**Figure 1:**
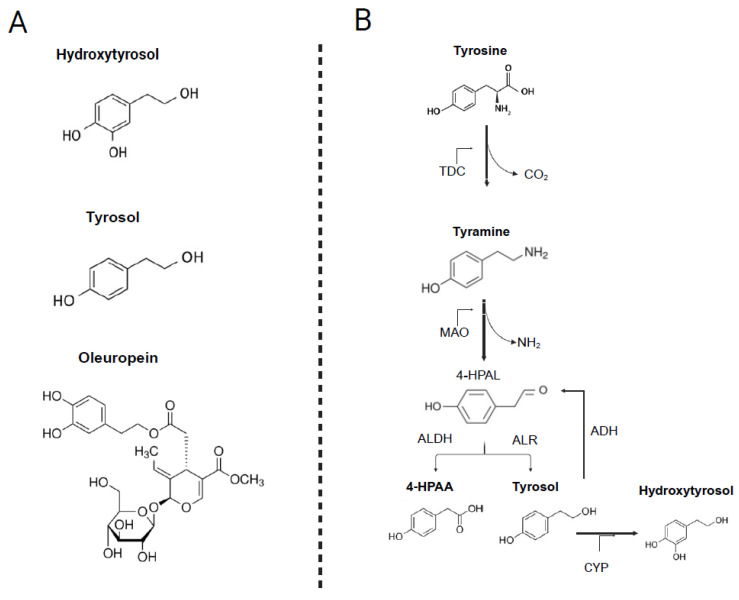
(**A**) Basic structure of hydroxytyrosol, tyrosol and oleuropein [47]. This figure presents the chemical structures of tyrosol (2-(4-hydroxyphenyl)-ethanol), hydroxytyrosol (3,4-dihydroxyphenylethanol), and oleuropein. Tyrosol features a benzene ring with a hydroxyl and an ethyl alcohol group, while HTyr includes an additional hydroxyl group, enhancing its antioxidant properties. Oleuropein, a complex phenolic compound, releases HTyr upon hydrolysis. (**B**) The metabolic pathway of endogenous tyrosol. This figure illustrates the biochemical pathway through which endogenous Tyr is produced from tyramine, a monoamine derived from the decarboxylation of the amino acid tyrosine by TDC. The pathway includes the deamination of tyramine by MAO to form the aldehyde intermediate 4-HPAL, which can be further processed by ALDH to yield 4-HPAA or reduced by ALR to generate Tyr. Additionally, the conversion of Tyr back to 4-HPAL is mediated by ADH. 4-HPAA: 4-hydroxyphenylacetic acid, 4-HPAL: 4-hydroxyphenylacetaldehyde, TDC: tyrosine decarboxylase, MAO: monoaminoxidase, ALDH: aldehyde dehydrogenase, ALR: aldehyde reductase, ADH: alcohol dehydrogenase, CYP: cytochrome P450, CYP2A6 and CYP2D6.

Tyrosol (Tyr) or 2-(4-hydroxyphenyl)-ethanol, is a simple phenolic alcohol composed of a benzene ring substituted with a hydroxyl group and an ethyl alcohol side chain. Its antioxidant activity primarily stems from its ability to donate hydrogen atoms. HTyr, a more potent antioxidant, is released through the hydrolysis of oleuropein (a complex phenolic compound) during olive maturation, storage, and processing, contributing substantially to the antioxidant properties of olive-derived products (Figure 1A).

### 2.2. Natural Dietary Sources

HTyr is one of the most abundant phenolic metabolites found in extra virgin olive oil, olives, and certain wines [21]. Tyrosol is likewise present in olives, olive oil, wine, and various herbal preparations [22].

### 2.3. Absorption and Metabolism of HTyr and Tyr

Current evidence indicates that HTyr and Tyr are mainly absorbed (in a dose-dependent manner) in the small intestine through passive diffusion, a process facilitated by their small molecular size and hydrophilicity. A smaller fraction is absorbed in the colon. Although several dietary polyphenols depend on membrane transporters, such as monocarboxylate transporters (MCTs), organic anion-transporting polypeptides (OATPs), glucose transporters (GLUTs), sodium-dependent glucose transporters (SGLTs), and peptide transporter 1 (PepT1), it has not been clearly demonstrated for HTyr or Tyr [23]. Peak plasma concentrations of HTyr metabolites typically occur within 30 min following ingestion of olive-derived supplements [24].

Although some phytochemicals can be absorbed directly in the stomach, most dietary polyphenols (typically present in food matrices as glycosides or conjugated to organic acids and sugars) require hydrolysis prior to absorption [25]. Gastric acidity promotes the hydrolysis of these compounds, thereby increasing their availability [26]. Once absorbed, both compounds undergo extensive phase I and phase II metabolism [27]. Phase I reactions involve the hydrolysis of secoiridoids such as oleuropein, releasing HTyr and Tyr [28]. Phase II metabolism occurs rapidly in enterocytes and hepatocytes and includes glucuronidation, sulfation, methylation, and oxidation, producing a wide range of conjugated metabolites. HTyr metabolism predominantly yields glucuronide and sulfate conjugates through the action of UGTs and SULTs. Additional pathways generate homovanillic acid (HVA) via catechol-O-methyltransferase (COMT), and 3,4-dihydroxyphenylacetic acid (DOPAC), which can be methylated to form HVA [29,30,31]. Other metabolites, including HT-acetate-4′-sulfate and mercapturates such as N-acetyl-5-S-cysteinyl-HT, have been detected, reflecting autoxidation and conjugation reactions [32,33]. Metabolomic studies indicate that most circulating HTyr exists in conjugated form, underscoring the biological importance of these metabolites [34,35]. A fraction of HTyr can also associate with circulating lipoproteins, which may contribute to its cardioprotective effects [28].

Some metabolites undergo enterohepatic recirculation, during which intestinal microbial enzymes may further deconjugate them before reabsorption [36]. Although microbial metabolism is important for HTyr/Tyr biotransformation, the detailed enzymatic pathways are discussed in Section 4.

HTyr and Tyr metabolites are distributed to multiple tissues, including the liver, kidneys, and brain, reflecting efficient systemic transport and the ability of certain metabolites to cross biological barriers, such as the blood–brain barrier [37]. HTyr has a short plasma half-life (approximately 8 min in humans) [38], while most excretion occurs via urine within the first 6 h after ingestion [39]. The European Food Safety Authority (EFSA) recommends an intake of at least 5 mg of HTyr per 20 g of olive oil to achieve clinically relevant antioxidant effects, particularly the protection of LDL from oxidative damage [40].

### 2.4. Endogenous Metabolism of Tyrosol

Endogenous Try is produced through the oxidative metabolism of tyramine, a biogenic amine generated by tyrosine decarboxylation via tyrosine decarboxylase (TDC) [31]. Tyramine is subsequently deaminated by monoamine oxidase (MAO), forming the aldehyde intermediate 4-hydroxyphenylacetaldehyde (4-HPAL). This intermediate may be oxidized by aldehyde dehydrogenase (ALDH) to yield 4-hydroxyphenylacetic acid (4-HPAA) or reduced by aldehyde reductase (ALR) to generate Tyr. Tyr may also be converted back to 4-HPAL via alcohol dehydrogenase (ADH) [30] (Figure 1B).

Once formed, Tyr undergoes rapid phase II metabolism in the liver, primarily sulfation and glucuronidation, producing metabolites such as 4-O-sulphate. These metabolites retain biological activity and exert protective effects, including the prevention of oxidized cholesterol-induced cell death [41,42]. Unlike HTyr, Tyr cannot undergo COMT-mediated methylation due to its molecular structure [43]; therefore, sulfation constitutes its main hepatic metabolic route. A portion of unabsorbed Tyr may also be converted to HTyr and other active metabolites by the gut microbiota, contributing further to its systemic bioactivity [44,45,46].

### 2.5. Factors Modulating Bioavailability and Interindividual Variability

The bioavailability of HTyr and Tyr varies considerably among individuals due to a wide range of physiological, genetic, dietary, and microbiome-related factors.

Dietary factors play a central role. The food matrix substantially influences absorption; HTyr delivered within extra virgin olive oil (EVOO) exhibits higher bioavailability than HTyr in aqueous solutions, likely due to improved micellar incorporation and protection from gastric oxidation [48,49]. Meal composition, including fat content, fiber, and the presence of other phenolic compounds, affects gastric emptying, intestinal transit, and the fraction of HTyr/Tyr reaching the colon [50].

The gut microbiota further contributes to interindividual variability by modulating the stability, transformation, and reabsorption of phenolic metabolites. Microbial enzymes participate in deglycosylation, deconjugation, and other biotransformations, influencing both the profile and abundance of circulating metabolites (discussed in detail in Section 4). Differences in microbial composition therefore create distinct metabolic phenotypes, or “metabotypes”, that may explain heterogeneity in cardiometabolic responses.

Genetic differences also contribute to variability in metabolic fate. Polymorphisms in metabolic enzymes, including CYP2A6, CYP2D6, UGT1A6, UGT2B7, and SULT1A1, modify the efficiency of oxidation, glucuronidation, and sulfation reactions, affecting systemic exposure to HTyr, Tyr, and their conjugates [24,51,52,53,54].

Additional modulators include age, sex, hepatic and renal function, metabolic status, and the use of medications such as proton pump inhibitors or antibiotics, which can influence digestive processes and the composition of gut microbiota [55]. Collectively, these factors shape the interindividual diversity in HTyr/Tyr absorption, metabolism, and biological activity.

## 3. The Role of Gut Microbiota in Atherosclerosis

### 3.1. Gut Microbiota Composition

The gut microbiota is a complex and dynamic ecosystem composed of thousands of microbial species that interact closely with the host, playing a central role in metabolic regulation and overall health [56]. Human microbiome studies have identified more than 2000 microbial species across 12 phyla [57], with *Firmicutes*, *Bacteroidetes*, *Actinobacteria*, *Proteobacteria*, and *Verrucomicrobia* representing the dominant phyla in the human gut [58]. Together, *Firmicutes* and *Bacteroidetes* account for over 90% of gut bacteria and are considered key components of a healthy microbiota [59]. The Firmicutes/Bacteroidetes (F/B) ratio is often used as a crude indicator of gut microbiota composition; however, this ratio varies widely across individuals and populations. A higher F/B ratio has been associated with obesity and, in some studies, with cardiometabolic disorders and cardiovascular risk [60]. Diet strongly influences microbial profiles: *Prevotella* tends to predominate in individuals consuming carbohydrate-rich diets, whereas *Bacteroides* is more abundant among those consuming diets high in animal protein and saturated fats [61,62].

Aging also affects gut microbiota composition, with older adults commonly exhibiting reduced microbial diversity and shifts toward facultative anaerobes, which may contribute to chronic inflammation and increased atherosclerosis risk [63,64]. Host-microbiota interactions are largely mediated by microbial metabolites and shaped by nutrient availability as well as microbial metabolic capacity [65,66]. Under physiological conditions, the colon’s low-oxygen environment supports strict anaerobes; however, diseases such as inflammatory bowel disease disrupt gut barrier integrity, allowing oxygen diffusion into the lumen and favoring facultative anaerobes.

The small intestine, characterized by acidic pH, antimicrobial peptides, and bile acids, is predominantly inhabited by *Lactobacillus*, *Streptococcus*, and *Proteobacteria*, whereas the colon harbors a much higher microbial density dominated by *Firmicutes* and *Bacteroidetes* [67]. Fermentation of dietary polysaccharides by colonic bacteria produces short-chain fatty acids (SCFAs), such as butyrate, which serve as energy substrates for colonocytes and maintain gut barrier integrity by limiting oxygen diffusion [68,69].

### 3.2. Gut Microbiota Functions Related to Host Metabolism

Gut microbes play a crucial role in degrading complex dietary polysaccharides that humans cannot digest [70]. This process relies on glycoside hydrolases and related enzymes that convert fibers into SCFAs (including acetate, propionate, and butyrate). SCFAs: (i) serve as an energy source for colonocytes, (ii) strengthen gut barrier integrity by reducing permeability, and (iii) regulate host metabolism, including inhibition of cholesterol synthesis via suppression of 3-hydroxy-3-methylglutaryl-CoA reductase (HMGR) [71]. *Bacteroides*, *Prevotella*, and SCFA-producing families such as *Lachnospiraceae* and *Ruminococcaceae* are key contributors to polysaccharide fermentation [72].

Microbial signals are essential for immune system maturation [70]. By presenting microbial antigens, they promote immune education and support the development of regulatory T cells (Tregs), which maintain immune tolerance [57]. SCFAs, particularly butyrate, modulate immune responses by influencing macrophages and dendritic cells (DCs), enhancing Treg differentiation and suppressing pro-inflammatory cytokines, while promoting anti-inflammatory mediators. Acetate contributes to the expansion of pre-existing colonic Tregs by activating free fatty acid receptor 2 (FFAR2) on T cells, whereas butyrate promotes de novo Treg differentiation by inhibiting histone deacetylase (HDAC) activity [73]. SCFAs also regulate Treg expansion via G protein-coupled receptor 109A(GPR109A) on DCs [74]. Additionally, butyrate suppresses the production of pro-inflammatory cytokines such as interleukin-12 (IL-12) and tumor necrosis factor alpha (TNF-α) by modulating nuclear factor kappa B (NF-κB) signaling, while enhancing anti-inflammatory cytokines including interleukin-10 (IL-10) and transforming growth factor-beta (TGF-β) [75].

**Figure 2:**
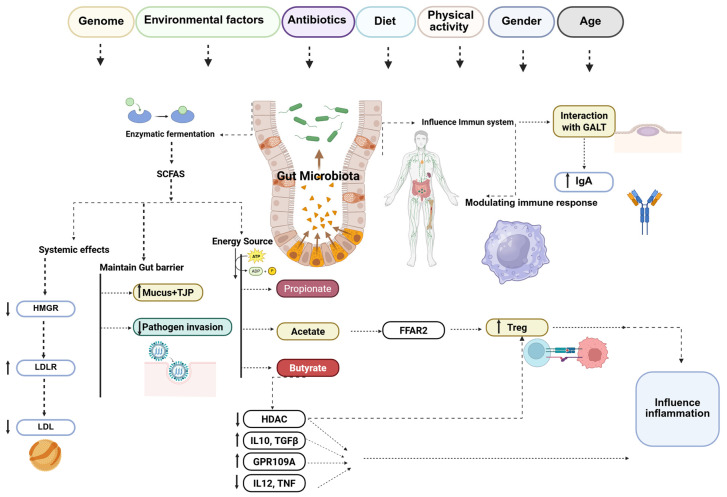
Factors influencing gut microbiota and mechanisms by which gut microbiota reduces inflammation, particularly through SCFAs. Multiple factors, including age, genetics, diet, environmental exposure, antibiotic use, physical activity, and sex/gender, influence the composition of gut microbiota. The microbiota plays a crucial role in immune system interactions, particularly with the GALT, where it stimulates IgA production and contributes to immune homeostasis. SCFAs such as acetate, propionate, and butyrate are key metabolites produced by microbial fermentation of dietary fibers. These SCFAs serve as energy substrates for colonocytes, strengthen gut barrier integrity by enhancing mucus production and TJP expression, and help prevent pathogen translocation. SCFAs also exert immunomodulatory effects. By activating Tregs via FFAR2 and inhibiting HDACs, they promote the expansion of Tregs. Butyrate, in particular, acts via GPR109A to induce anti-inflammatory cytokines (IL-10, TGF-β) and suppress pro-inflammatory cytokines (IL-12, TNFα), thereby attenuating intestinal and systemic inflammation. Moreover, SCFAs influence lipid metabolism. They have been associated with decreased expression of HMGR and plasma LDL levels, along with increased LDLR expression, highlighting a potential cardiometabolic benefit. FFAR2: free fatty acid receptor 2, GALT: gut-associated lymphoid tissue, GPR109A: G protein-coupled receptor 109A, HDAC: histone deacetylases, HMGR: 3-hydroxy-3-methyl-glutaryl-CoA reductase, IgA: immunoglobulin A, IL: interleukin, LDLR: low-density lipoprotein receptor, TGF-β: transforming growth factor beta, TNF-α: tumor necrosis factor alpha, TJP: tight junction proteins, Treg: regulatory T cell. Note: Arrows indicate direction of change (↓ decrease; ↑ increase).

Specific bacteria, such as *Bacteroides fragilis*, further enhance Treg function through polysaccharide A (PSA) and activation of Toll-like receptor 2 (TLR2) [76]. Gut microbes also support gut barrier integrity by stimulating the production of mucus and tight junction proteins (TJPs), thereby limiting pathogen translocation. Moreover, they interact with gut-associated lymphoid tissue (GALT), promoting the production of immunoglobulin A (IgA), a key component of mucosal immunity that neutralizes harmful pathogens within the gut [65,77,78] (Figure 2).

### 3.3. Gut Microbiota Dysbiosis and Atherosclerosis

Dysbiosis, characterized by reduced microbial diversity and the expansion of specific taxa, has been strongly linked to metabolic diseases [79,80,81,82], chronic inflammation, immune dysregulation [16,83,84,85,86], and coronary artery disease [87,88]. These alterations often modulate individual responses to dietary or pharmacological interventions.

Modern lifestyle factors, including increased hygiene, widespread antibiotic use, westernized diets, and physical inactivity, have substantially altered the human microbial ecosystem [89].

Microbial imbalances promote cholesterol accumulation [90], strengthen pro-oxidative and pro-inflammatory signaling pathways [91]. Intervention studies using probiotics and microbiota-derived metabolites suggest potential strategies for CVD prevention and management [92,93].

One of the earliest indicators of a gut-vessel axis was the detection of bacterial DNA within atherosclerotic plaques [94]. Atherogenesis is influenced by (i) microbial metabolites such as trimethylamine N-oxide (TMAO) and phenylacetylglutamine (PAGln), (ii) microbial regulation of bile acids, (iii) immune modulators including lipopolysaccharides (LPSs) and Tregs, (iv) oxidized LDL, (v) SCFAs, and (vi) polyamines [14]. These factors activate macrophages within both the arterial wall and the intestinal mucosa, promoting plaque initiation and progression [95]. LPS and other large molecules can enter systemic circulation when intestinal barrier integrity is compromised, contributing to chronic inflammation [96].

Foam cell formation and impaired efferocytosis also play central roles in linking dysbiosis to atherosclerosis. Under physiological conditions, Mer proto-oncogene tyrosine kinase (MerTK)-mediated efferocytosis clears apoptotic cells and supports the resolution of inflammation through HDL-mediated reverse cholesterol transport (RCT). When efferocytosis fails, foam cells accumulate and amplify inflammation. Microbiota-derived factors such as LPS and TMAO exacerbate these dysfunctions by sustaining inflammation, enhancing LDL oxidation, and altering macrophage cholesterol handling, collectively promoting plaque instability [96,97].

**Figure 3:**
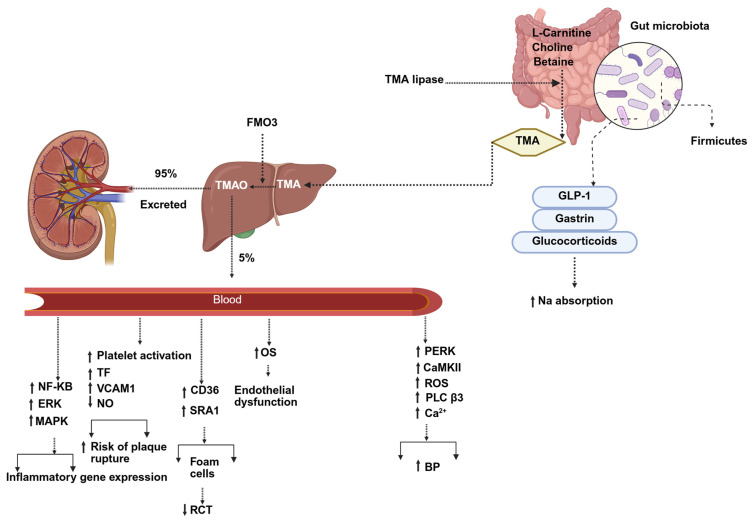
TMA and Atherosclerosis: proposed mechanisms. Gut microbiota, particularly Firmicutes, convert dietary precursors as L-carnitine, choline, and betaine into TMA via TMA lyase. TMA is absorbed into the bloodstream and transported to the liver, where it is oxidized by FMO3 into TMAO. Although 95% of TMAO is excreted by the kidneys, elevated circulating levels has been implicated in increased risk of ASCVD. TMAO contributes to plaque instability and rupture by enhancing platelet activation and upregulating TF and VCAM1, while simultaneously reducing NO bioavailability. In macrophages, TMAO promotes foam cell formation through the upregulation of CD36 and SRA1, impairs RCT, and exacerbates inflammation via activation of MAPK, ERK, and NF-kB signaling pathways. Endothelial dysfunction is further driven by TMAO-induced oxidative stress. Additionally, TMAO has been linked to elevated blood pressure through activation of signaling pathways involving PERK, ROS, CaMKII, PLC β3, and intracellular Ca^2+^. BP: blood pressure, FMO3: flavin-containing monooxygenase 3, GLP-1: glucagon-like peptide-1, ERK: extracellular signal-regulated kinase, MAPK: mitogen-activated protein kinase, NO: nitric oxide, NF-κB: nuclear factor-κB, PERK: protein kinase R-like endoplasmic reticulum kinase, SR-A1: class A1 scavenger receptor, ROS/CaMKII/PLC β3/Ca^2+^: reactive oxygen species/calmodulin-dependent protein kinase II/phospholipase C β3/Ca^2+^, RCT: reverse cholesterol transport, TMA: trimethylamine, TMAO: trimethylamine N-oxide, TF: tissue factor, VCAM1: vascular cell adhesion molecule 1. Note: Arrows indicate direction of change (↓ decrease; ↑ increase).

Among the most well-characterized microbiota-derived pro-atherogenic metabolites is trimethylamine (TMA), generated from dietary precursors such as L-carnitine, choline, and betaine, through the activity of TMA lyases (enzymes predominantly expressed by *Firmicutes*) [98]. TMA is subsequently oxidized in the liver to form TMAO by flavin-containing monooxygenase 3 (FMO3) [99]. Elevated TMAO levels, which are modulated by diet, kidney function, and microbiota composition, are consistently associated with increased CVD risk, adverse outcomes, and mortality [99,100,101,102,103,104,105]. TMAO promotes atherogenesis by upregulating macrophage scavenger receptors CD36 and scavenger receptor class A1 (SR-A1), enhancing foam cell formation and impairing RCT [90]. It also enhances platelet activation, increases endothelial adhesion molecule expression (including tissue factor (TF) and vascular cell adhesion molecule-1 (VCAM-1)) [106], and may reduce nitric oxide (NO) bioavailability [107,108]. Furthermore, TMAO activates inflammatory signaling pathways, including the mitogen-activated protein kinase (MAPK), extracellular signal-regulated kinase (ERK), and NF-κB [109,110]. In hypertension, TMAO activates protein kinase RNA-like endoplasmic reticulum kinase (PERK) and downstream ROS-CaMKII-PLCβ3-Ca^2+^ pathways (reactive oxygen species (ROS), calmodulin-dependent protein kinase (CaMKII), phospholipase C β3 and intracellular calcium (Ca^2+^)) [111]. Additionally, other gut microbiota-derived metabolites, including gastrin, glucocorticoids, and glucagon-like peptide-1 (GLP-1), influence sodium absorption and contribute to blood pressure regulation [112] (Figure 3).

Phenylacetylglutamine (PAGln), a microbial metabolite of phenylalanine, promotes platelet activation and thrombosis through α- and β-adrenergic receptor signaling, similar to catecholamines. Its effects can be attenuated by β-blockers such as carvedilol, highlighting potential therapeutic relevance [14].

Gut microbes play a central role in bile acid biotransformation. Bacterial 7-α-dehydroxylase converts primary bile acids into secondary bile acids, deoxycholic acid (DCA) and lithocholic acid (LCA), which activate farnesoid X receptor (FXR) and Takeda G protein-coupled receptor 5 (TGR5) to regulate lipid and glucose metabolism [113,114].

Western diets promote dysbiosis and excessive secondary bile acid production, weakening the gut barrier and facilitating inflammatory signaling through Toll-like receptor 4 (TLR4) pathways [115].

Gut microbiota also shapes T cell differentiation across multiple subsets, including T-helper 1 (Th1), Th2, Th17, Follicular helper T (Tfh), Treg, and unconventional populations such as γδ T cells, natural killer T cells (NKTs), and mucosal-associated invariant T (MAIT) [116,117].

SCFAs modulate T cell activation, cytokine production, and cytotoxicity in a context-dependent manner [118,119,120,121,122].

Butyrate-producing bacteria (e.g., *Roseburia* and *Eubacterium*) are reduced in atherosclerosis. Butyrate, a key microbial metabolite, suppresses inflammatory signaling (e.g., histone deacetylases (HDACs) and NF-κB), strengthens the gut barrier integrity, and promotes hepatic β-oxidation, collectively mitigating plaque development [115,123,124].

Beyond their roles in immune regulation and barrier integrity, SCFAs also exert significant metabolic effects. SCFAs exert complex effects on lipid metabolism. Acetate promotes hepatic lipogenesis [125], whereas propionate inhibits cholesterol synthesis via HMGR suppression [126]. A lower acetate/propionate ratio may therefore attenuate lipid synthesis [127]. Propionic acid supplementation reduced cholesterol levels and atherosclerotic lesions in mice and improved lipid profiles in humans [128]. SCFA production varies by bacterial strain [129,130,131,132], yet overall SCFA profiles remain relatively stable due to functional redundancy across taxa.

SCFAs may also lower cholesterol by upregulating sterol regulatory element-binding protein 2 (SREBP2), increasing LDL receptor expression and enhancing LDL clearance [133]. Probiotic and prebiotic interventions upregulate hepatic SREBP2 expression in animal models [134,135], suggesting a mechanistic link to lipid homeostasis.

Polyamines, including putrescine, spermidine, and spermine, are synthesized by gut microbes as well as obtained from the diet [136]. They promote autophagy, reduce inflammation, and support cardiovascular protection [137,138,139]. Clinical trials show that increasing polyamine production through probiotics or arginine-enriched foods improves endothelial function and reduces atherosclerosis risk [140].

Probiotic strains, such as *Plantarum* (10^9^ CFU/day), improve lipid profiles in animals by upregulating hepatic CYP7A1 and enhancing bile acid excretion [141,142]. In humans, probiotics and synbiotics improve lipid profile, blood pressure, and metabolic parameters, particularly in individuals with type 2 diabetes mellitus (T2DM) [143]. These findings underscore the potential of microbiota modulation as a strategy to influence lipid metabolism, a key determinant of atherosclerosis risk.

## 4. Bidirectional Interactions Between Polyphenols (with a Focus on HTyr/Tyr) and the Gut Microbiota

HTyr and Tyr health benefits arise from (i) direct actions in the gastrointestinal tract, (ii) systemic effects of absorbed and conjugated metabolites, and (iii) microbiota-driven transformations that modify bioactivity and bioavailability [144]. This bidirectional interplay contributes to the heterogeneity observed in polyphenol intervention studies and highlights the gut microbiota as a promising therapeutic and precision-nutrition target in chronic diseases, including atherosclerosis.

### 4.1. Microbial Biotransformation of Hydroxytyrosol and Tyrosol

The gut microbiota extensively metabolizes polyphenols into lower molecular weight metabolites, which have distinct absorption profiles and biological activities [145]. Several bacterial enzymes involved in polyphenol metabolism have been identified, such as those from *Eubacterium ramulus*, which catalyze polyphenolic ring fission, generating bioactive metabolites with altered health properties [146]. Microbial enzymes, including β-glucosidase, esterases, α-rhamnosidase, and β-glucuronidase, contribute to deglycosylation, deconjugation, and structural modification of polyphenols in the colon [17]. For HTyr/Tyr, several transformations occur: (1) oleuropein and its aglycone are hydrolyzed by microbial esterases to release HTyr, (2) unabsorbed Tyr can be converted into HTyr and other low-molecular-weight compounds, (3) microbial reactions may include ring fission, dehydroxylation, demethylation, reduction, and isomerization, producing metabolites with variable antioxidant, anti-inflammatory, or signaling properties [45,46,147].

These metabolic products can act locally within the gut (enhancing epithelial integrity, reducing LPS translocation, and modulating immune responses) or can be absorbed systemically, where they may contribute to the anti-atherogenic benefits of HTyr and Tyr.

However, microbial enzymatic pathways involved remain incompletely characterized, underscoring the need for further research to map strain-specific transformations [148].

### 4.2. Effects of HTyr/Tyr on Gut Microbiota Composition

Human studies directly assessing the specific effects of HTyr on gut microbiota are still limited; however, evidence from EVOO and polyphenol-rich interventions provides valuable insights. Polyphenols promote the growth of beneficial taxa such as *Lactobacillus*, *Bifidobacterium* and *Akkermansiaceae*, which exhibit anti-inflammatory and cardioprotective activities [149,150,151]. Such taxa contribute to improved gut barrier function, enhanced production of SCFAs (especially butyrate) and reduced intestinal inflammation. SCFAs strengthen epithelial integrity, regulate immune responses, decrease endotoxemia, and support lipid and glucose metabolism along the gut-heart axis [152,153,154,155].

Conversely, polyphenols can inhibit potentially pathogenic bacteria, including *Clostridium* spp., thereby attenuating gut-derived inflammatory stimuli involved in cardiometabolic disorders [13]. Therefore, the anti-atherogenic properties of EVOO phenolics may be partially mediated through their ability to promote a beneficial gut microbiota profile. However, these modulatory effects are not uniform, highlighting the critical influence of interindividual factors such as microbiome differences, a concept explored in the following sections.

### 4.3. Host and Dietary Factors Influencing Polyphenol-Microbiota Interactions

The interaction between HTyr/Tyr and the gut microbiota is strongly influenced by dietary context and host physiology. Dietary fiber interactions and food matrix components determine the fraction of phenolics that reaches the colon; that is, they influence polyphenol bioaccessibility (release from the food matrix) and bioavailability (absorption and utilization) [156,157,158]. These interactions determine how much of an ingested polyphenol becomes available for microbial metabolism and subsequent systemic effects. High-fiber matrices may delay absorption and increase colonic delivery, whereas refined or low-fiber matrices may favor early absorption and reduce microbial exposure [50].

In the specific case of HTyr and Tyr, the food matrix (e.g., oil, bakery products, extracts), co-ingested nutrients, and meal composition influence gastric emptying, micellar incorporation, and intestinal transport, thereby shaping the fraction that reaches the colon versus the systemic circulation. It has been reported that compared with HTyr and secoiridoids, oleuropein exhibits greater stability during digestion, resulting in higher bioavailability based on urine excretion of HTyr metabolites. Furthermore, oleuropein, due to its glycoside structure, reaches the colon largely unaltered, generating a more diverse range of microbial metabolites [50].

The gut microbiota further modulates polyphenol stability through enzymatic reactions, including deglycosylation, sulfation, glucuronidation, and ring cleavage, which affect their metabolic pathway and health effects [156].

Medication use (e.g., proton pump inhibitors, antibiotics) and inter-individual differences in digestive function further modify these interactions, introducing additional variability in HTyr/Tyr-microbiota crosstalk in real-world dietary settings [159,160,161].

Gut physiological factors such as transit time and pH strongly influence the composition and metabolic activity of the gut microbiota; consequently, digestive function and intestinal transit modulate the microbial metabolism of polyphenols [162].

Together, these factors determine the metabolic fate of HTyr/Tyr and shape downstream vascular and cardiometabolic effects.

### 4.4. Interindividual Variability and the Concept of “Metabotypes”

The concept of “metabotypes” has emerged to describe distinct interindividual patterns of microbial metabolite production in response to the same polyphenol intake. Classic examples include differences in urolithin production from ellagitannins and O-DMA formation from daidzein [163]. In the context of EVOO phenolics, metabolomic studies demonstrate considerable diversity in urinary and plasma metabolites following standardized olive polyphenol intake [164]. Urolithins, for instance, are more readily absorbed than their precursors and mediate the health benefits of foods like berries and nuts [165].

These findings support the existence of distinct “HTyr/Tyr metabotypes”, in which individuals differ in their ability to generate specific conjugated and microbial metabolites. Such variability is influenced by age and sex, genetics, diet, medication use, lifestyle, and baseline gut microbiota composition [55]. As a result, individuals exposed to similar HTyr/Tyr doses may exhibit very different circulating metabolite profiles and, consequently, divergent cardiometabolic responses.

Although current evidence clearly implicates the microbiota in EVOO polyphenol biotransformation, human data specifically characterizing the microbial metabolism of oleuropein, Tyr, and their derivatives remain limited. Future studies should integrate detailed phenolic profiling, microbiome sequencing, and cardiometabolic phenotyping to (i) identify HTyr/Tyr metabotypes, (ii) link these metabotypes to vascular and inflammatory outcomes, and (iii) determine whether tailoring HTyr/Tyr dosing or formulation to microbiota profiles can enhance atherosclerosis prevention strategies.

## 5. Mechanistic Insights into Cardiometabolic Protection

HTyr and Tyr, two major phenolic compounds found in EVOO, exert multiple biological effects that collectively contribute to cardiovascular protection. Their actions include modulation of endothelial function, oxidative stress, inflammatory pathways, thrombosis, lipid and glucose metabolism, and mitochondrial activity. Together, these mechanisms, individually and synergistically, may attenuate the initiation and progression of atherosclerosis.

Most mechanistic insights derive from in vitro and animal models. These studies provide biological plausibility but may not fully reflect human physiology, habitual dietary exposures, or interindividual differences in gut microbiota. Therefore, the mechanistic data summarized below should be interpreted alongside evidence from human intervention studies (Section 5.5).

### 5.1. Endothelial Protective and Anti-Thrombotic Effects

Endothelial, smooth muscle, and peripheral blood cells are chronically exposed to elevated levels of toxic molecules, proinflammatory cytokines, chemokines, eicosanoids (prostaglandins and leukotrienes) and autoantibodies [166].

In non-inflammatory human endothelial cells, HTyr at concentrations ranging from 0.1 to 100 µM did not affect endothelial nitric oxide synthase (eNOS) gene promoter activity, eNOS activity, or NO bioavailability [167]. However, in ECV304 cells, a model of endothelial dysfunction associated with type 2 diabetes, HTyr (10 μM, 48 h) significantly increased endothelin-1 (ET-1) expression, enhanced eNOS phosphorylation, and increased nitric oxide production, thereby partially counteracting endothelial dysfunction induced by high glucose and free fatty acids [168].

Moreover, HTyr and its metabolites have been shown to reduce the secretion of several adhesion molecules, including E-selectin, P-selectin, intercellular adhesion molecule 1 (ICAM-1), and VCAM-1 in human aortic endothelial cells, which may limit leukocyte recruitment and early atherosclerotic lesion formation [169].

Tyrosol exerts similar protective effects by reducing HDL oxidation and improving its cholesterol efflux capacity [170]. Its antiatherogenic properties may also stem from the inhibition of leukotriene B4 production, a mediator known to impair endothelial function [171]. Furthermore, Samuel et al. reported that Tyr promotes myocardial protection under ischemic stress by activating proteins associated with cellular survival and longevity, suggesting potential cardioprotective and anti-aging benefits [172].

HTyr exhibits significant anti-thrombotic properties by reducing platelet aggregation both in vitro and ex vivo. Dell’Angi et al. identified inhibition of cyclic adenosine monophosphate phosphodiesterase (cAMP-PDE) as a key mechanism underlying this effect [173]. Additionally, Medina-Martinez et al. demonstrated that HTyr impairs platelet activation by inhibiting both cAMP-PDE and cyclic guanosine monophosphate phosphodiesterase (cGMP-PDE), thereby reinforcing its antiplatelet activity [174]. In healthy elderly individuals, HTyr supplementation has been associated with reduced vascular calcification, a recognized marker of advanced atherosclerosis [175].

Collectively, these findings indicate that HTyr and Tyr preserve endothelial function by modulating key proteins involved in proliferation and migration, reducing leukocyte adhesion, and attenuating platelet activation [147], key early events in atherothrombosis.

### 5.2. Antioxidant and Mitochondrial Protective Effects

HTyr is the principal contributor to the EFSA health claim that “olive oil polyphenols contribute to the protection of blood lipids from oxidative stress” when at least 5 mg HTyr and its derivatives are consumed per 20 g of olive oil [40]. This claim reflects its potent antioxidant properties, which have been linked to improvements in LDL oxidation, HDL functionality, and vascular homeostasis [176].

ROS activate proto-oncogenes and pro-inflammatory genes by increasing intracellular calcium [177]. HTyr’s antioxidant activity is primarily due to its ortho-hydroxyl functional groups, which enable electron donation and the formation of stable hydrogen bonds with phenoxyl radicals, effectively neutralizing oxidative species [178,179]. In the study of Perrone et al., acute intake of 25 g of phenol-rich EVOO significantly reduced oxidative stress biomarkers, including oxidized LDL and malondialdehyde (MDA), while upregulating antioxidant genes such as catalase and superoxide dismutase-1 (SOD-1) in healthy volunteers [180]. In vitro and in vivo studies consistently show that HT reduces ROS and MDA and increases NO bioavailability, supporting its vascular protective effects [181].

Mitochondrial dysfunction and excessive production of mitochondrial reactive oxygen species (mtROS) are key contributors to endothelial dysfunction and the development of ASCVD. HT has been shown to protect mitochondria by reducing mtROS, preserving mitochondrial membrane potential, and promoting mitochondrial biogenesis. In endothelial cells activated by phorbol 12-myristate 13-acetate (PMA), HTyr significantly decreased O_2_^.−^ production, lipid peroxidation, and mitochondrial membrane depolarization, while increasing SOD activity and mtDNA content. It also upregulated key regulators of mitochondrial biogenesis, including peroxisome proliferator-activated receptor gamma coactivator-1α (PGC-1α), nuclear respiratory factor-1 (NRF-1), and mitochondrial transcription factor A (TFAM), thereby improving mitochondrial functionality [182].

In H9c2 cardiomyocytes with elevated xanthine/xanthine oxidase (X/XO) activity, HTyr (0.1 and 10 μg/mL for 24 h) reduced intracellular ROS levels and activated protective pathways via p44/42-MAPK and Hsp27 through c-Jun [183]. Similarly, in porcine endothelial cells exposed to H_2_O_2_, HTyr (10–50 μM) attenuated excessive ROS accumulation, activated AMP-activated protein kinase (AMPK) and FOXO3a, and upregulated catalase (CAT) [184].

Sirtuin 1 (SIRT1), a key regulator of vascular NO production and vascular aging, is often downregulated under oxidative stress and inflammation conditions, contributing to endothelial dysfunction [185,186]. HTyr (50 μM) activates the SIRT1–Nrf2 axis, promoting nuclear accumulation of nuclear factor-E2-related factor 2 (Nrf2) and increasing heme oxygenase-1 (HO-1) expression, thereby enhancing cellular defenses and wound healing capacity [187,188,189,190]. Nrf2 regulates several antioxidant genes and plays a central role in protecting vascular endothelial cells from oxidative injury, a pathway that HTyr appears to reinforce.

HTyr also exhibits antimicrobial activity against a wide range of microorganisms, including *Escherichia coli*, *Candida albicans*, *Clostridium perfringens* and *Streptococcus mutans,* as well as probiotic strains such as *Lactobacillus acidophilus* and *Bifidobacterium bifidum* [174,191]. HTyr has demonstrated antiparasitic properties [39] and can inhibit ROS production triggered by microbial biofilms. However, not all studies confirm these antimicrobial effects [192]. These mixed findings, along with HTyr’s capacity to modulate microbial communities, suggest that it may influence gut microbiota composition and barrier integrity, indirectly affecting cardiometabolic risk.

Tyrosol, despite having a weaker radical-scavenging capacity than HTyr, is a stable and effective cellular antioxidant [193]. It protects L6 skeletal muscle cells from H_2_O_2_-induced oxidative damage by preventing cell death through modulation of ERKs, c-Jun N-terminal kinase (JNK), and p38 MAPK pathways, while also enhancing ATP production [194]. Tyrosol also shows antibacterial activity against pathogens associated with intestinal and respiratory infections [174], possibly through inhibition of bacterial ATP synthase and disruption of microbial energy metabolism [195]. Overall, these findings indicate that, although HTyr generally displays stronger direct antioxidant potency due to its catechol structure, Tyr still provides meaningful protection through its stability, mitochondrial-supporting actions, and modulation of stress-response kinases.

### 5.3. Anti-Inflammatory Pathways

Inflammation is a vital defense mechanism against cellular damage and pathogen threats, largely regulated by the NF-κB signaling pathway. HTyr exhibits potent anti-inflammatory properties by modulating oxidative stress and interfering with key inflammatory mediators. Specifically, HTyr inhibits enzymes such as cyclooxygenase (COX) and lipoxygenase (LOX), as well as arachidonic acid (AA) metabolism, thereby reducing the production of pro-inflammatory cytokines, including TNF-α and IL-1β [196,197].

HTyr at a concentration of 50 μM demonstrates strong anti-inflammatory effects in LPS-stimulated RAW264.7 macrophages by inhibiting NF-κB [198]. Similarly, in J774 murine macrophages, HTyr suppressed inducible nitric oxide synthase (iNOS) and COX-2 expression by counteracting NF-κB, STAT-1α, and IRF-1 activation, key transcription factors upregulated in LPS-induced oxidative stress [191].

In Apo E^−^/^−^ mice, daily administration of HTyr (10 mg/kg/day for 16 weeks) significantly reduced aortic atherosclerotic lesions and systemic inflammation, as reflected by decreased levels of C-reactive protein (CRP), TNF-α, IL-1β, and IL-6, and increased anti-inflammatory IL-10 [199]. The protective effect of HTyr was attributed to inhibition of p38MAPK phosphorylation and NF-κB activation in hepatic tissue, thereby reducing inflammation-driven atherogenesis.

HTyr also shows systemic anti-inflammatory effects in models of acute inflammation. In a mouse model of endotoxemia, oral HTyr (80 mg/kg/day) administered before LPS injection significantly reduced plasma TNF-α in Balb/c mice [200], indicating potential to mitigate acute systemic inflammatory responses.

In human studies, particularly in patients with chronic coronary artery syndrome (CCAS), supplementation with HTyr-enriched olive oil has been associated with improved vascular function. Parameters such as flow-mediated dilation (FMD), coronary flow reserve (CFR), and pulse wave velocity (PWV) improved, while MDA, ox-LDL, triglycerides (TGs), proprotein convertase subtilisin/kexin type 9 (PCSK9), and CRP decreased [201]. In contrast, a short-term study in healthy individuals consuming HTyr-enriched olive mill wastewater extract (5–25 mg/day for 7 days) did not observe significant changes in inflammatory biomarkers (hs-CRP, IL-6, IL-8, IL-10, IL-17, monocyte chemoattractant protein 1 (MCP-1), TNF-α, and vascular endothelial growth factor (VEGF)) [202]. These contrasting findings highlight the influence of baseline cardiovascular risk, dose, and intervention duration on the detectability of anti-inflammatory effects.

HTyr also modulates immune responses beyond classical inflammation pathways. It may reduce immune recognition of allergenic molecules, potentially mitigating allergic responses [203]. Olive oil polyphenols, including HTyr, inhibit mast cell degranulation and histamine release through both immune-mediated and non-immune mechanisms [204].

**Figure 4:**
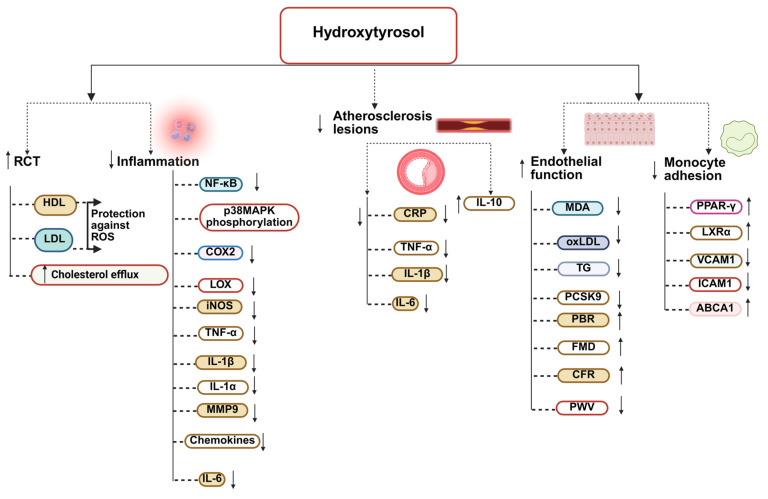
Anti-inflammatory effects of HTyr and atherosclerosis: proposed mechanisms. The diagram illustrates the proposed mechanisms by which HTyr exerts anti-inflammatory effects that contribute to the attenuation of atherosclerosis lesions. HTyr plays a role in enhancing endothelial function, reducing inflammation and oxidative stress, promoting reverse cholesterol transport, and decreasing monocyte adhesion to the vascular endothelium. Key pathways involved include the modulation of inflammatory markers (e.g., CRP, TNF-α, IL-6), oxidative stress markers (e.g., malondialdehyde, ox-LDL), and adhesion molecules (e.g., ICAM-1, VCAM-1). ABCA1: ATP binding cassette subfamily a member 1, CFR: coronary flow reserve, COX-2: cyclooxygenase-2, CRP: C-reactive protein, FMD: flow-mediated dilation, HDL: high-density lipoprotein, ICAM-1: intercellular adhesion molecule 1, IL-1β: interleukin-1 beta, IL-1α: interleukin-1 alpha, IL-6: interleukin-6, iNOS: inducible nitric oxide synthase, LOX: lipoxygenase, LXRα: liver X receptor alpha, MDA: malondialdehyde, MMP-9: matrix metallopeptidase 9, NF-κB: nuclear factor kappa B, ox-LDL: oxidized low-density lipoprotein, PBR: perfused boundary region, PCSK9: proprotein convertase subtilisin/kexin type 9, PPARγ: peroxisome proliferator-activated receptor gamma, PWV: pulse wave velocity, RCT: reverse cholesterol transport, ROS: reactive oxygen species, TG: triglyceride, TNF-α: tumor necrosis factor-alpha, VCAM-1: vascular cell adhesion molecule 1. Note: Arrows indicate direction of change (↓ decrease; ↑ increase).

Recent studies suggest that HTyr influences endothelial activation and monocyte adhesion, the key early steps in atherogenesis. By activating the PPARγ/LXRα pathway, HTyr upregulates ABCA1 expression, promoting cholesterol efflux, reducing foam cell formation, and inhibiting monocyte adhesion to vascular endothelial cells [205]. Tyrosol may exert related effects through modulation of CD14 and upstream inflammatory signaling [206] (Figure 4).

While direct head-to-head comparisons are scarce, in vitro studies indicate that HTyr is the major anti-inflammatory constituent of olive extracts, producing more pronounced reductions in cytokine and chemokine release from macrophages. Tyr also attenuates LPS-induced inflammation by suppressing NF-κB and p38/ERK MAPK signaling and promoting CD14 shedding in macrophages and endothelial models, although generally at higher concentrations or with slightly lower potency [198,206,207].

### 5.4. Effects on Lipid and Glucose Metabolism

The metabolic effects of HTyr have received increasing attention due to their relevance for obesity, dyslipidemia, and diabetes, all of which are tightly linked to atherosclerosis. HTyr has been reported to improve blood lipid profiles by lowering total cholesterol, TG, and LDL levels in experimental models [208], although human data remain heterogeneous [209]. HTyr prevents TNF-α-mediated suppression of adiponectin (a hormone with anti-diabetic, anti-inflammatory, and anti-atherogenic properties) by preserving its expression in adipocytes [210].

HTyr regulates adipogenesis-associated genes by inhibiting preadipocyte differentiation, promoting lipolysis, and inducing apoptosis in differentiating preadipocytes. Mechanistically, HTyr downregulates SREBP-1c, a key transcription factor in lipogenesis, and delays cell cycle progression [211,212]. In rat models, HTyr ameliorates hyperlipidemia by modulating genes involved in adipocyte maturation and differentiation while suppressing lipid accumulation [213].

In diet-induced obese mice, 10 weeks of HTyr administration improved glucose homeostasis, reduced chronic inflammation, and decreased hepatic steatosis. Mechanistically, HTyr attenuated high-fat diet (HFD)-induced endoplasmic reticulum (ER) stress and insulin resistance by regulating insulin signaling in the JNK/IRS pathways. HTyr also downregulated hepatic SREBP-1 and lipogenic genes, including acetyl-CoA carboxylase 1 (ACC), fatty acid synthase (FAS), and stearoyl-CoA desaturase 1 (SCD1) [214]. In white adipose tissue (WAT) of HFD-fed mice, HTyr modulated NF-κB, Nrf2, SREBP-1c, and PPAR-γ, and their downstream targets, thereby attenuating inflammation, enhancing antioxidant defenses, and modulating lipogenesis [215]. HTyr also exerts calorie restriction-like effects in muscle, brain, fat tissue, and kidneys, primarily via sirtuin activation [216].

In terms of lipoprotein metabolism, enrichment of LDL and HDL with HTyr and Tyr (up to 25 μM) enhances resistance to oxidation and promotes RCT. HTyr metabolites (HTyr -sulfate, homovanillic acid sulfate, and glucuronide conjugates) have been detected bound to HDL particles, potentially contributing to antioxidant protection and preventing oxidative modifications of ApoA-I and other HDL-associated proteins [31,170].

HTyr also exerts significant effects on glucose metabolism. In diabetic rats, HTyr supplementation reduced intestinal disaccharidase and lipase activities, increased antioxidant enzyme activities (SOD, CAT, GPx), and elevated reduced glutathione, thereby decreasing oxidative stress and improving glycemic control [217].

Tyrosol has likewise been shown to improve hyperglycemia by modulating key enzymes of carbohydrate metabolism in streptozotocin-induced diabetic rats. It may help manage diabetes by exerting anti-inflammatory effects in the liver and pancreas [218] and by inhibiting ER stress-induced apoptosis in pancreatic β-cells [219]. Tyrosol has also been reported to downregulate lipid synthesis in primary rat hepatocytes [220].

**Figure 5:**
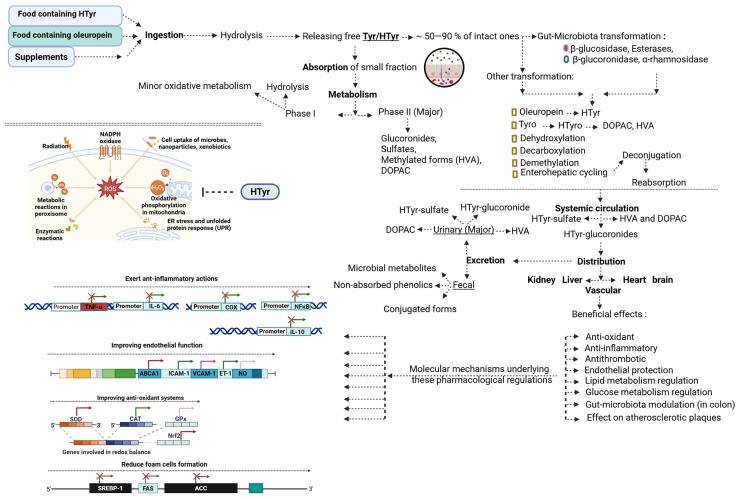
Integrated cardiometabolic mechanisms of HTyr and Tyr in atherosclerosis. Schematic representation of the main pathways, from absorption and metabolism to downstream actions, through which HTyr and Tyr may attenuate atherosclerosis. ABCA1: ATP-binding cassette transporter A1; ACC: acetyl-CoA carboxylase; CAT: catalase; COX: cyclooxygenase; DOPAC: 3,4-dihydroxyphenylacetic acid; ER: endoplasmic reticulum, ET: Endothelin-1, FAS: fatty acid synthase; GPx: glutathione peroxidase; HTyr: hydroxytyrosol; HVA: homovanillic acid; ICAM-1: intercellular adhesion molecule-1; IL-6: interleukin-6; IL-10: interleukin-10; NF-κB: nuclear factor kappa-light-chain-enhancer of activated B cells; NO: nitric oxide; Nrf2: nuclear factor erythroid 2-related factor 2; ROS: reactive oxygen species; SOD: superoxide dismutase; SREBP-1: sterol regulatory element-binding protein-1; TNF-α: tumor necrosis factor-alpha; Tyr: tyrosol; VCAM-1: vascular cell adhesion molecule-1. Note: Arrows indicate direction of change (↓ decrease; ↑ increase); blunt-ended lines (⊣) indicate inhibition.

Overall, preclinical data support a role for HTyr and Tyr in improving lipid and glucose metabolism, but the translation of these effects to humans appears to depend on dose, duration, baseline metabolic status, and potentially gut microbiota composition (Figure 5).

### 5.5. Evidence from Human Clinical Studies

#### 5.5.1. Overall Effects on Cardiometabolic Biomarkers

As summarized in Table 1, human clinical trials generally report favorable, though heterogeneous, effects on cardiometabolic biomarkers. The most consistent findings are improvements in oxidative stress markers, even at moderate doses, while lipid-lowering and anti-inflammatory effects are more variable and often dependent on the population’s baseline metabolic risk.

A closer analysis reveals a distinct hierarchy of responsiveness. The most robust and consistent effects of HTyr and Tyr supplementation are observed on oxidative stress biomarkers, which are often improved even in healthy subjects at moderate doses. Lipid-lowering (and possibly vascular functional benefits) are more variable but frequently reported, particularly in cohorts with elevated cardiometabolic risk. In contrast, significant effects on systemic inflammation and glycemic control are less consistently demonstrated, likely requiring higher doses, longer durations, or more specific patient metabotypes to detect.

A recent meta-analysis combining evidence from human intervention studies on oleuropein, HTyr, and Tyr further supports their cardiometabolic benefits. The pooled evidence showed significant reductions in total cholesterol, TG, and fasting insulin, indicating modest improvements in lipid metabolism and insulin regulation. Benefits appeared more pronounced in individuals with cardiometabolic risk or in non-Mediterranean populations. In addition, subgroup analyses indicated that supplements were more effective than enriched foods in reducing TG and insulin levels, suggesting that baseline metabolic status and dietary context may influence responsiveness [221]. 

**Table 1:** Summary of human clinical studies investigating the effects of Hydroxytyrosol, Tyrosol and Oleuropein on oxidative stress, inflammation and cardiometabolic markers.

Study, Year	Country	Study Design	Population	Duration (Weeks)	Intervention	N		Control	Sex (M/F)	Measured Outcomes
						Intervention	Control			
Vázquez-Velascoet al., 2011 [222]	Spain	Randomized controlled trial, crossover	Healthy subjects	3	Enriched sunflower: 10–15 g/d (45–50 mg/d HTyr)	22	22	Non-enriched sunflower oil	M/F	↑ ARE (PON-1 activity), ↓ ox-LDL, ↓ sVCAM-1, ↔ TC, ↔ HDL, ↔ LDL, ↔ TG, ↔ BMI, ↔ BW
de Bock et al., 2013 [223]	New Zealand	Randomized controlled trial, crossover	Overweight subjects	12	Olive leaf extract capsules: (51.1 mg/d oleuropein and 9.7 mg/d HTyr)	22	23	Safflower oil	M	↑ IL-6, ↔ IL-8, ↔ TNF-α, ↔ CRP, ↔ ox-LDL, ↔ TC, ↔ HDL, ↔ LDL, ↔ TG, ↔ BW, ↔ BMI, ↔ BP
Crespo et al., 2015 [202]	Spain	Randomized controlled trial, Latin square design	Healthy subjects	1	Enriched olive mill wastes water extract: 5 or 25 mg/d HTyr	21	21	Placebo (maltodextrin)	M/F	↔ IL-6, ↔ IL-8, ↔ IL-10, ↔ IL-17, ↔ MCP-1, ↔ TNF-α, ↔ VEGF, ↔ hs-CRP, ↔ Iso, ↔ ox-LDL, ↔ TXB2, ↔ TC, ↔ HDL, ↔ LDL, ↔ TG, ↔ BW, ↔ BMI, ↔ BP, ↔ BF
Filip et al., 2015 [224]	Poland	Randomized controlled trial, parallel	Postmenopausal and osteopenic	52	Supplementation: 250 mg/d olive extract (100 mg/d oleuropein) + 1000 mg Ca	32	32	Placebo + 1000 mg Ca	F	↔ hs-CRP, ↔ IL-6, ↓ TC, ↓ LDL, ↔ HDL, ↓ TG, ↔ BW, ↔ BMI
Colica et al., 2017 [225]	Italy	Randomized controlled trial, crossover	Healthy subjects	3	Supplementation: (15 mg/d HTyr)	28	28	Placebo	M/F	↑ TAS, ↑ Thiols, ↑ SOD-1, ↓ MDA, ↔ TC, ↔ HDL, ↔ LDL, ↔ TG, ↔ FBG, ↔ Ins, ↔ ox-LDL, ↓ BW, ↔ WC, ↔ WHR
Lockyer et al., 2017 [226]	New Zealand	Randomized controlled trial, crossover	Pre-hypertensive subjects	6	Olive leaf extract: (136.2 mg/d oleuropein and 6.4 mg/d HTyr)	60	60	Placebo	M	↓ IL-8, ↔ ox-LDL, ↔ CRP, ↔ ICAM, ↔ VCAM, ↔ P-s, ↔ E-s, ↔ IL-1β, ↔ IL-6, ↔ IL-10, ↔ TNF-α, ↓ TC, ↓ HDL, ↓ LDL, ↓ TG, ↔ FBG, ↔ Ins, ↔ HOMA-IR, ↔ QUICKI, ↔ BP
Araki et al., 2019 [227]	Japan	Randomized controlled trial, parallel	Pre-diabetic subjects	12	Olive leaf tea: (324 mg/d oleuropein and 12 mg/d HTyr)	28	29	Olive leaf tea: (24 mg/d oleuropein and 3 mg/d HT)	M/F	↓ TG, ↓ LDL, ↔ HDL, ↓ FBG, ↔ Ins, ↔ HOMA-IR, ↔ HbA1c, ↔ BW, ↔ WC
Conterno et al., 2019 [228]	Italy	Randomized controlled trial, parallel	Hypercholesterolaemic subjects	8	Enriched biscuits with olive pomace: (15.39 mg/d HTyr and its derivatives)	34	28	Control biscuits	M/F	↔ BW, ↔ BMI, ↔ WC, ↔ BP, ↔ HDL, ↔ TC, ↔ LDL, ↔ TG, ↔ Apo A1, ↔ Apo B, ↔ FBG, ↔ Ins, ↔ CRP, ↔ ox-LDL, ↔ F2 Iso
Dinu et al., 2021 [229]	Italy	Randomized controlled trial, crossover	Healthy subjects	8.5	Olive pâté supplementation: (30 mg/d HTyr)	19	19	Placebo	M/F	↑ Nrf-2, ↓ MCP-1, ↔ IL-1 ra, ↔ IL-6, ↔ IL-8, ↔ IL-10, ↔ IL-12, ↔ IL-17, ↔ TNF-α, ↔ VEGF, ↓ TC, ↔ HDL, ↓ LDL, ↔ TG, ↔ HOMA-IR, ↔ Ins, ↔ FBG, ↔ BW, ↔ BMI
Stevens et al., 2021 [230]	Netherlands	Randomized controlled trial, parallel	Overweight/obese subjects	8	Olive leaf extract supplementation: (83.5 mg/d oleuropein)	39	38	Placebo	M/F	↔ ox-LDL, ↔ BP, ↔ HR, ↔ BW, ↔ BMI, ↔ TG, ↔ LDL, ↔ HDL, ↔ TC, ↔ FBG, ↔ Ins
Fytili et al., 2022 [231]	Greece	Randomized controlled trial, parallel	Overweight/obese subjects	24	Supplementation: (15 mg/d HTyr)	9	11	Placebo	F	↓ BW, ↓ BF
Horcajada et al., 2022 [232]	Belgium	Randomized controlled trial, parallel	Subjects with knee pain	26	Olive leaf extract supplementation: (100 mg/d oleuropein)	59	59	Placebo	M/F	↔ PGE2, ↔ IL-8, ↔ TNF-α
Binou et al., 2023 [233]	Greece	Randomized controlled trial, parallel	Overweight/obese subjects with T2DM	12	Enriched whole wheat bread: (32.5 mg/d HTyr)	30	30	Control whole wheat bread	M/F	↔ HbA1c, ↓ FBG, ↓ TC, ↓ LDL, ↔ HDL, ↔ TG, ↔ Ins, ↔ Leptin, ↔ BP, ↔ Adiponectin, ↔ hs-CRP, ↔ TNF-α, ↔ ox-LDL, ↔ BW, ↔ BMI, ↔ WC, ↓ BF
Ikonomidis et al., 2023 [201]	Greece	Randomized controlled trial, crossover	Subjects with chronic CAS	4	Supplementation: (Olive oil + 10 mg/d HTyr)	30	30	Olive oil	M/F	↑ FMD, ↑ PWV, ↑ PBR, ↑ CFR, ↓ MDA, ↓ PCSK9, ↓ CRP, ↓ ox-LDL, ↓ TG, ↔ TC, ↔ LDL, ↔ HDL, ↔ BP
Naranjo et al., 2024 [234]	Spain	Randomized controlled trial, parallel	Subjects with acute ischemic stroke	6	Supplementation: (15 mg/d HTyr)	4	4	Placebo	M	↔ FBG, ↔ HbA1c, ↔ TC, ↔ LDL, ↓ HDL, ↔ TG, ↓ NO, ↔ IL-6, ↔ TBARS, ↔ BMI, ↔ BP
Imperatrice et al., 2024 [235]	Netherlands	Randomized controlled trial, parallel	Postmenopausal women	12	Olive leaf extract supplementation: (100 mg/d oleuropein)	29	31	Placebo	F	↓ TG, ↔ TC, ↔ LDL, ↔ HDL, ↔ BF
Pinckaers et al., 2025 [236]	Netherlands	Randomized controlled trial, parallel	Older males	5	Olive leaf extract supplementation: (100 mg/d oleuropein)	20	20	Placebo	M	↓ LDL, ↔ HDL, ↔ TG, ↔ TC
Moratilla-Rivera et al., 2025 [237]	Spain	Randomized controlled trial, parallel	Overweight subjects with prediabetes	16	Supplementation: (15 mg/d HTyr)	24	25	Placebo	M/F	↓ ox-LDL, ↓ PCO, ↓ 8-OHdG, ↓ IL-6, ↔ *TAS, ↔ *GPx, ↔ TC, ↔ LDL, ↔ HDL, ↔ TG, ↔ BW, ↔ BMI
Haidari et al., 2025 [238]	Iran	Randomized controlled trial, parallel	Obese subjects	8	Olive leaf extract supplementation: (100 mg/d oleuropein)	31	32	Placebo	F	↓ MDA, ↔ TAS

Note: Arrows indicate the direction of significant changes between intervention and control groups: ↓ denotes a significant decrease, ↑ denotes a significant increase (*p* < 0.05), and ↔ indicates no significant difference. Abbreviations: N, number of participants; Apo A1, apolipoprotein A1; Apo B, apolipoprotein B; ARE, arylesterase activity; BF, body fat; BMI, body mass index; BP, blood pressure; BW, body weight; Ca, calcium; CAS, chronic coronary artery syndrome; CFR, coronary flow reserve; CRP, C-reactive protein; DBP, diastolic blood pressure; E-s: E-selectin, FBG, fasting blood glucose; F2 Iso, F2-isoprostanes; FMD, flow-mediated dilation; HbA1c, hemoglobin A1c; HDL, high-density lipoprotein; HR, heart rate; HOMA-IR, homeostasis model assessment of insulin resistance; hs-CRP, high-sensitivity C-reactive protein; ICAM, intercellular adhesion molecule; IL-1β, interleukin-1 beta; IL-1 ra, interleukin-1 receptor antagonist; IL-6, interleukin-6; IL-8, interleukin-8; IL-10, interleukin-10; IL-12, interleukin-12; IL-17, interleukin-17; Ins, insulin; LDL, low-density lipoprotein; MCP-1, monocyte chemoattractant protein-1; MDA, malondialdehyde; Nrf-2, nuclear factor erythroid 2–related factor 2; NO, nitric oxide; 8-OHdG, 8-hydroxy-2-deoxyguanosine; ox-LDL, oxidized low-density lipoprotein; PBR, perfused boundary region; PCSK9, proprotein convertase subtilisin/kexin type 9; PGE2, prostaglandin E2; PCO, protein carbonyls; P-s: P-selectin, PWV, pulse wave velocity; QUICKI, quantitative insulin sensitivity check index; sVCAM-1, soluble vascular cell adhesion molecule-1; SOD-1, superoxide dismutase-1; T2DM, type 2 diabetes mellitus; TAS, total antioxidant status; TBARS, thiobarbituric acid reactive substances; TC, total cholesterol; TG, triglycerides; TNF-α, tumor necrosis factor alpha; TXB2, Thromboxane B2; VCAM, vascular cell adhesion molecule; VEGF, vascular endothelial growth factor; WC, waist circumference; WHR, waist-to-hip ratio; M/F, male/female. * Intervention prevented decline in the HTyr group compared to the decline observed in the control group (*p* < 0.01).

#### 5.5.2. Dose–Response Considerations

Across the included clinical trials, establishing a clear dose–response relationship for HTyr, Tyr, and oleuropein is challenging due to substantial variability in (i) doses (e.g., 10–15 mg/day vs. ≥30 mg/day HTyr; 83–136 mg/day vs. 324 mg/day oleuropein), (ii) food matrices (enriched oils, bakery products, extracts, capsules), (iii) phenolic composition and how these compounds are reported across trials, (iv) intervention duration, and (v) population.

In general, low-to-moderate HTyr doses (~15–30 mg/day) tend to improve oxidative stress biomarkers (e.g., reducing MDA, ox-LDL, PCO, 8-OHdG, and increasing TAS, thiols, Nrf2-related markers) even over relatively short periods, suggesting that antioxidant pathways respond at relatively low exposure levels. Lipid-lowering outcomes (e.g., decreasing TC, LDL, TG) are more frequently observed with higher oleuropein doses or in metabolically compromised participants (prediabetes, overweight/obesity, T2DM, pre-hypertension, postmenopausal). Effects on inflammatory cytokines and glycemic control remain inconsistent and largely dose-independent, with many trials (regardless of dose) reporting no significant changes in IL-6, IL-8, CRP, TNF-α, insulin, or HOMA-IR.

Overall, current evidence supports the presence of potential “threshold effects”: antioxidant improvements appear achievable at lower doses and shorter durations, whereas clinically meaningful changes in lipid or metabolic outcomes may require higher doses, longer interventions, or populations with elevated baseline cardiometabolic risk.

A summary of the human intervention studies on HTyr, Tyr, and oleuropein, including study design, population, intervention details, and primary outcomes, is presented in Table 1, while key mechanistic outcomes from preclinical studies are summarized in Table 2.

#### 5.5.3. Risk of Bias (RoB2) Assessment

**Figure 6:**
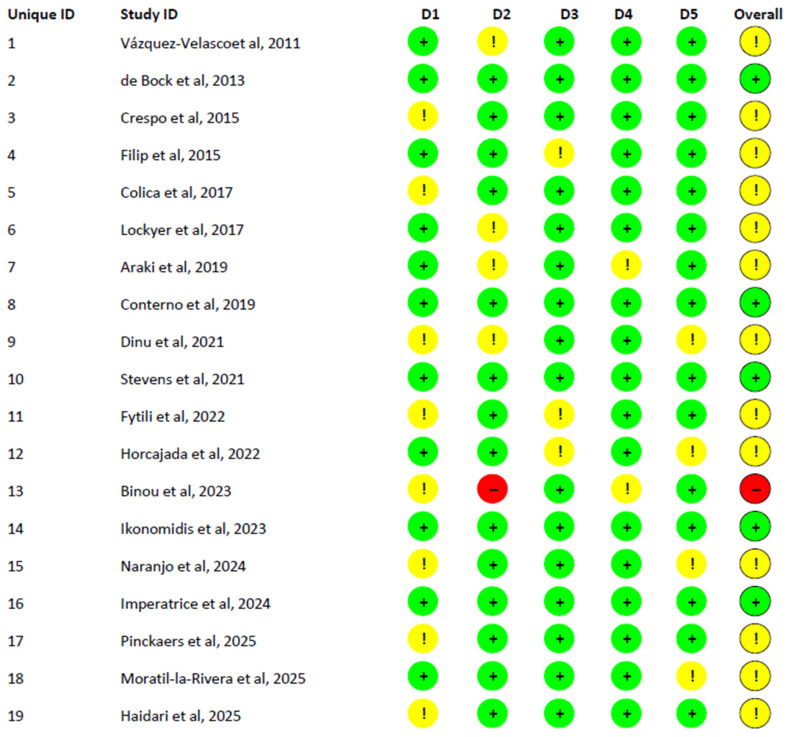
Risk of bias assessment for the included randomized controlled trials (RoB2) [201,202,222,223,224,225,226,227,228,229,230,231,232,233,234,235,236,237,238]. Risk of Bias (RoB2): The overall risk of bias for each randomized controlled trial was assessed using the Cochrane RoB2 tool. 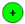 Green (Low Risk): The study is methodologically sound and provides trustworthy results. 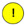 Yellow (Some Concerns): The study has some methodological limitations, but the results are likely credible. 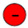 Red (High Risk): The study has significant methodological flaws, and its results should be interpreted with caution. Note: Each circle represents the judgment for a specific domain: D1 = randomization process; D2 = deviations from the intended interventions; D3 = missing outcome data; D4 = measurement of the outcome; D5 = selection of the reported result. Green indicates low risk, yellow indicates some concerns, and red indicates high risk of bias. An overall risk-of-bias judgment is also provided for each study, based on the combined domain assessments.

Risk of bias was assessed for all 19 randomized controlled trials using the Cochrane RoB2 tool. Overall, the methodological quality was moderate. Among the included trials, 5 have a low risk of bias, 13 were rated as having some concerns in at least one domain, and 1 study was classified as high risk due to issues in deviations from intended interventions (D2) and the selection of reported results (D5). The most frequent sources of potential bias across studies were the randomization process (D1) and deviations from intended interventions (D2), while missing outcome data (D3) and outcome measurement (D4) were generally assessed as low risk. Detailed domain-level judgments and overall ratings are presented in Figure 6.

### 5.6. Summary of Clinical and Mechanistic Benefits

Collectively, HTyr and Tyr exhibit multifunctional protective effects that counteract key processes in atherogenesis. Their antioxidant, anti-inflammatory, endothelial-protective, antithrombotic, and metabolic activities act synergistically to preserve vascular function and reduce cardiometabolic risk. Clinical trials, though heterogeneous, broadly support small-to-moderate improvements in oxidative stress, lipid profile, and vascular function, particularly in individuals with elevated baseline risk.

However, important knowledge gaps remain regarding optimal dosing, intervention duration, formulation (food vs. supplement), and interindividual variability linked to gut microbiota composition and HTyr/Tyr metabotypes. These elements likely contribute to the heterogeneity of clinical responses and should be systematically incorporated into future trial designs.

### 5.7. Summary of Preclinical Evidence (In Vitro and Animal Studies)

Preclinical studies in rodent and cell models largely support the biological plausibility that HTyr and Tyr modulate oxidative stress, inflammation, endothelial function, lipid and glucose metabolism, and immune responses. In ApoE^−^/^−^ mice and hyperlipidemic rabbits, HTyr reduces atherosclerotic lesion area, improves lipid profiles, and lowers systemic inflammatory markers, while in diet-induced obesity and metabolic syndrome models, it attenuates hepatic steatosis, ER stress, and insulin resistance. In vitro, HTyr and Tyr enhance ABCA1-mediated cholesterol efflux, protect endothelial mitochondria, inhibit LDL oxidation, and regulate NF-κB-driven inflammatory responses in macrophages and adipocytes (Table 2). Nonetheless, these models often employ supraphysiological doses and short exposure periods that exceed typical dietary intake, and they generally fail to account for human gut microbiota diversity, underscoring the need for cautious translation to clinical settings.

## 6. The HTyr/Tyr-Gut Microbiota-Atherosclerosis Axis

The interaction between HTyr, Tyr, and the gut microbiota forms a dynamic axis that links dietary polyphenols with cardiovascular protection. After ingestion, only a fraction of HTyr and Tyr are absorbed in the small intestine; the remainder reaches the colon, where microbial enzymes convert them into diverse low-molecular-weight metabolites. These derivatives (mainly glucuronidated, sulfated, and methylated forms) retain antioxidant and anti-inflammatory activities that act both locally in the gut and systemically. Thus, the gut microbiota not only determines the bioavailability and metabolic fate of EVOO phenolics but also responds to them, creating a feedback loop that influences host metabolism and vascular health.

**Figure 7:**
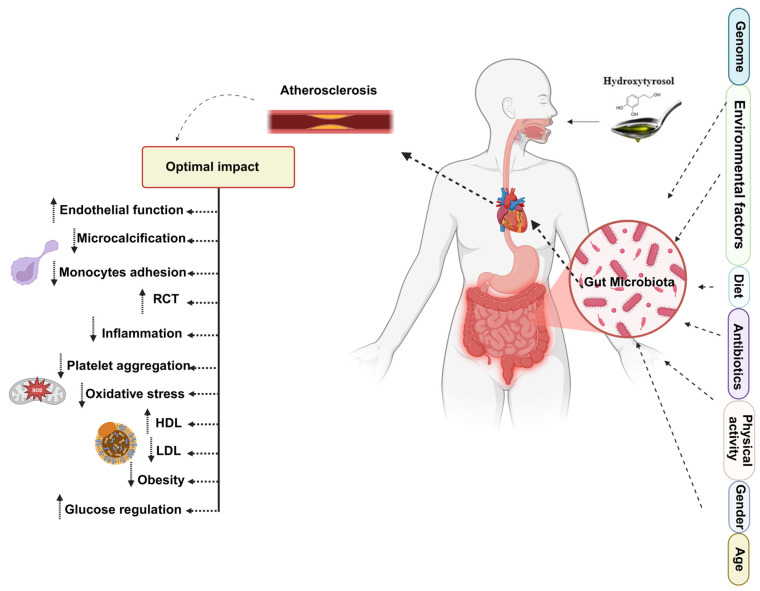
Interrelationship between HTyr and gut microbiota in the prevention of atherosclerosis. HTyr contributes to cardiovascular health both directly and indirectly through its interaction with gut microbiota, which is influenced by factors such as age, diet, and lifestyle. This synergetic relationship helps support endothelial function, reduce inflammation and oxidative stress, and regulate lipid and glucose metabolism, factors that collectively reduce the risk of atherosclerosis. Despite growing evidence, the precise mechanisms underlying the interaction between HTyr and gut microbiota remain incompletely understood. Note: Arrows indicate direction of change (↓ decrease; ↑ increase).

Despite growing interest, data on the microbial metabolism of oleuropein and other EVOO-derived polyphenols in humans remain scarce, and results concerning their detection in biofluids are inconsistent [154,239]. The metabolic fate of EVOO polyphenols depends on both dietary factors and the individual’s gut microbiota composition [240]. In this context, HTyr/Tyr should be viewed as part of a broader “polyphenol-microbiota axis”, in which many metabolites retain significant biological activity, influencing cellular signaling pathways and contributing to their health-promoting effects [241] (Figure 7).

HTyr has been shown to preserve intestinal morphology and mitigate oxidative stress-induced damage in mice [242]. Wang et al. demonstrated that HTyr upregulates the expression of MUC2, a key gene involved in maintaining intestinal barrier integrity. This upregulation indirectly promotes epithelial renewal and supports the regeneration of the intestinal barrier [243]. By reinforcing tight junctions, mucus production, and local antioxidant defenses, HTyr and its metabolites may limit microbial translocation and systemic exposure to pro-atherogenic mediators such as LPS and TMAO, thereby indirectly attenuating vascular inflammation and plaque progression.

From a translational perspective, these findings suggest that the cardiovascular effects of HTyr and Tyr may differ markedly between individuals depending on their baseline microbiota composition, capacity to generate specific metabolites (e.g., HTyr conjugates, DOPAC, HVA), and integrity of the intestinal barrier. In practice, this means that comparable dietary intakes of EVOO phenolics may yield distinct ‘responders’ (might experience the full health benefits) and ‘non-responders’ (might experience little to no benefit, perhaps due to differences in their metabolism, gut bacteria, or genetics) phenotypes, driven by differences in microbial metabolism, gut ecology, and host genetics. Such variability is rarely studied in current clinical trials, which typically do not stratify participants by gut microbiota features or metabolite profiles, but it is likely critical for designing personalized HTyr/Tyr-based interventions.

Although current evidence supports the role of gut microbiota in polyphenol biotransformation and highlights the cardioprotective properties of HTyr and its metabolites (Figure 7), the precise mechanisms connecting HTyr, gut microbiota, and atherosclerosis remain to be fully elucidated. Key unresolved questions include: (i) which microbial taxa and enzymes generate the most cardioprotective HTyr/Tyr metabolites; (ii) how these metabolites interact with vascular, immune, and metabolic pathways in vivo; and (iii) the extent to which diet, medications, and host genetics modulate these interactions over time. Interindividual variations in gut microbiota composition, dietary habits, and the chemical forms of HTyr and Tyr may significantly modulate these effects.

Future studies should integrate targeted and untargeted metabolomics, microbiome sequencing, and detailed cardiometabolic phenotyping to dissect the HTyr/Tyr-gut microbiota-atherosclerosis axis. Incorporating microbiota-based stratification (metabotypes) into controlled dietary interventions with long-term clinical endpoints will be essential for translating promising mechanistic findings into effective, microbiome-informed cardiovascular prevention strategies.

## 7. Challenges, Translational Limitations, and Future Perspectives

### 7.1. Methodological, Bioavailability, and Dose-Relevance Limitations

Despite encouraging mechanistic and clinical findings, several methodological factors limit the translation of HTyr/Tyr research into clinical practice. Human trials show substantial heterogeneity in dose, food matrix [49], formulation, and phenolic composition, with some studies reporting only total phenols rather than quantifying HTyr, Tyr, or oleuropein individually. This lack of standardization complicates dose–response evaluation and hinders identification of minimally effective intakes.

Most human trials are short in duration, involve small sample sizes, and often enroll healthy or mildly at-risk participants, a design that may underestimate effects on glycemic control, adiposity, inflammation, or vascular remodeling. Variability in food matrix (oils, extracts, bread, biscuits, pâté) further influences intestinal absorption, metabolism, and systemic availability of conjugated metabolites.

Although improvements in oxidative biomarkers (e.g., TAS, ox-LDL, MDA, PCO) are consistent, effects on inflammatory cytokines, glycemic indices, and adiposity remain variable, possibly due to insufficient duration, lower doses, or differences in participants’ metabolic status.

Preclinical models often employ supraphysiological doses that exceed those attainable through habitual diets, making it difficult to define physiologically relevant supplemental doses. Although HTyr doses of 10–30 mg/day and oleuropein doses of 80–136 mg/day show acceptable tolerability, long-term safety data and potential nutrient or drug interactions remain incomplete.

### 7.2. Biological Differences Between HTyr and Tyr, Safety, and Potential Interactions

Although HTyr and Tyr share similar structures, HTyr consistently demonstrates greater radical-scavenging capacity, stronger Nrf2 activation, more pronounced mitochondrial protection, and more effective suppression of inflammatory mediators. In contrast, Tyr (thought to be less potent) appears more stable and may exert more sustained intracellular antioxidant effects, including anti-inflammatory actions via MAPK and TLR4/CD14 signaling pathways. These qualitative differences are rarely examined in direct comparative human studies, representing an important evidence gap. Notably, at higher or pharmacological doses, oleuropein and HTyr may also exhibit pro-oxidant activity, a mechanism proposed to underlie antiproliferative effects observed in cancer models [244,245].

Polyphenols, including HTyr and Tyr, may also interact with dietary or endogenous proteins through hydrogen bonding, hydrophobic interactions, and, in some cases, covalent binding. Such interactions can modify protein structure, solubility, and susceptibility to digestive enzymes, with potential implications for nutrient digestibility and enzymatic activity under conditions of high polyphenol intake. Additionally, polyphenols possess iron-chelating properties, which, while beneficial in states of iron overload, could reduce intestinal iron absorption when consumed in excess, potentially affecting iron homeostasis in susceptible individuals [246].

Potential interactions with cardiometabolic medications (e.g., antiplatelet agents, anticoagulants, statins, antihypertensives, antidiabetic drugs) remain largely unexplored. Given the antithrombotic, vasomodulatory, and antioxidant properties of HTyr, the mechanistic overlap with common cardiovascular drugs warrants careful evaluation in future trials.

In particular, the mild antiplatelet aggregation effects of HTyr may theoretically potentiate the action of aspirin or anticoagulants (e.g., warfarin), as suggested by in vitro and ex vivo platelet aggregation studies. Also, HTyr had a weaker effect compared to hydroxytyrosol acetate [247,248].

Similarly, the vasodilatory and endothelial-restorative actions of HTyr may interact with antihypertensive agents (e.g., calcium-channel blockers), although no clinical interaction studies exist [181].

Given that both HTyr and Tyr improve insulin sensitivity and oxidative stress, theoretical interactions with metformin or GLP-1 analogues may also exist, but human data are lacking [212,249].

Potential interactions with other dietary supplements (such as other polyphenol-rich extracts, omega-3 fatty acids, probiotics, or nutraceutical antioxidants) should also be considered, as these may amplify or attenuate the biochemical effects of HTyr/Tyr through shared antioxidant, anti-inflammatory, or gut microbiota-modulating pathways [250,251].

Overall, while HTyr and Tyr appear safe at typical nutritional and supplemental doses, rigorous clinical trials are needed to define potential pharmacokinetic and pharmacodynamic interactions, especially in individuals taking multiple cardiometabolic medications.

Although no clinical studies have demonstrated cardiovascular harm from high intakes of HTyr or Tyr, very high or pharmacological doses may induce pro-oxidant effects, alter iron homeostasis, or affect protein-nutrient interactions, which could theoretically have unfavorable consequences. These potential risks remain speculative and require dedicated evaluation in future clinical studies.

### 7.3. Interindividual Responses and Microbiota-Driven Variability

Interindividual variability constitutes one of the most important but underexplored limitations in current HTyr/Tyr research. Gut microbiota composition, microbial enzymatic capacity, dietary habits, age, sex, genetic, medication use, and metabolic status all influence the transformation, absorption, and systemic circulation of HTyr/Tyr metabolites.

Emerging evidence supports the presence of distinct “HTyr/Tyr metabotypes,” reflecting differential microbial conversion into conjugates (e.g., HTyr-sulfate, glucuronides), DOPAC, HVA, or other metabolites. These metabotypes likely determine which individuals respond favorably to HTyr/Tyr interventions. Thus, identical dietary HTyr/Tyr intake may produce distinct ‘responder’ and ‘non-responder’ phenotypes, driven by interindividual differences in microbiota composition and metabolism, an aspect rarely studied in current clinical trials but essential for personalized interventions.

### 7.4. Priority Gaps for Future Human Studies

To improve translation into clinical practice, future research should incorporate:

- Dose-ranging randomized controlled trials using standardized and chemically characterized phenolic compositions.
- Longer intervention durations capable of assessing glycemic control, adiposity, vascular remodeling, and cardiometabolic outcomes.
- Parallel profiling of circulating metabolites (metabolomics, lipidomics) to confirm bioavailability and metabolic pathways in humans.
- Detailed microbiome sequencing to identify HTyr/Tyr metabotypes and characterize responder vs. non-responder phenotypes.
- Controlled lifestyle factors, including dietary background, to reduce confounding from macronutrient and micronutrient patterns, fiber intake, or other polyphenols.
- Systematic evaluation of drug-nutrient interactions, especially in populations taking cardiometabolic medications.
- Comparative studies of HTyr vs. Tyr to clarify their differential potency and specific mechanisms.

Collectively, addressing these gaps will enhance the evidence base and support the development of microbiota-informed, personalized HTyr/Tyr interventions for cardiometabolic health.

## 8. Conclusions

Overall, current evidence shows that HTyr, Tyr, and the gut microbiota participate in interconnected pathways relevant to atherosclerosis. Gut microbes influence the metabolic fate and biological activity of HTyr/Tyr, while these phenolics can, in turn, modulate microbial composition and intestinal barrier integrity.

Numerous studies link gut microbiota features to cardiometabolic processes, and preclinical findings support the biological plausibility of HTyr’s vascular and metabolic actions. Microbiota composition and metabolic capacity likely determine individual responsiveness to HTyr/Tyr, underscoring the need for personalized approaches.

Inconsistencies across human trials largely reflect heterogeneity in dose, duration, phenolic composition, study populations, and analytical techniques. Nevertheless, the role of HTyr in protecting lipids from oxidation is sufficiently recognized that EFSA recommends consuming EVOO providing at least 5 mg of HTyr per 20 g of olive oil [40].

Looking ahead, key research priorities include defining dose–response thresholds, identifying HTyr/Tyr metabotypes, standardizing microbiota and phenolic profiling methods, and conducting long-term trials with clinically relevant endpoints. Integrating HTyr/Tyr supplementation with microbiota-informed precision nutrition strategies may offer a promising route toward individualized cardiovascular prevention, a hypothesis that warrants rigorous validation in future human studies.

## Figures and Tables

**Table 2:** Summary of preclinical evidence from animal and in vitro studies evaluating the biological effects of Hydroxytyrosol, Tyrosol and Oleuropein on atherosclerosis, metabolic dysfunction, oxidative stress, inflammation, and gut microbiota-related mechanisms.

Model	Intervention	Mechanistic Pathway	Main Results	Reference
In vivo				
Wistar rats (female), healthy	HTyr or secoiridoids (SECs) at 5 mg/kg/day, 21 days, oral diet	Proteomic modulation of cardiovascular tissues ↓ endothelial cell proliferation and migration ↓ vascular occlusion pathways ↓ pathways associated with heart failure	↓ proteins related to endothelial proliferation, migration, and vascular occlusion (aorta) ↓ proteins linked to heart failure (heart tissue) SEC had stronger effects than HTyr	[147]
Rats (MI model induced by LAD ligation)	Tyr 5 mg/kg/day, 30 days	↑ SIRT1 ↑ Akt-P ↑ eNOS-P ↑ FOXO3a-P	↓ infarct size (32% vs. 48%) ↓ cardiomyocyte apoptosis ↑ EF ↑ fractional shortening ↑ eNOS-P ↑ Akt-P ↑ FOXO3a-P ↑ SIRT1	[172]
Male ApoE^−^/^−^ mice	HTyr 10 mg/kg/day orally, 16 weeks	Regulated AMPK/SREBP2 ↑ ABCA1, apoAI, SR-BI ↓ p-p38 ↑ AMPK activation ↓ NF-κB ↓ SREBP2/PCSK9 ↑ LDLR, apoAI, ABCA1	↓ Atherosclerotic lesions ↓ serum TG, TC, LDL ↓ hepatic TG and TC ↑ HDL ↓ serum CRP, TNF-α, IL-1β, IL-6 and ↑ IL-10	[199]
Balb/c mice (40 animals, 5 groups) with LPS-induced systemic inflammation	HTyr (40 or 80 mg/kg), or 80 mg/kg for 5 days; oral gavage. Two doses given during fasting (at 8 h and 24 h). LPS injection given 1 h after last gavage; sacrifice 2 h later.	Anti-inflammatory and antioxidant effects (↓ COX2, ↓ TNF-α, ↓ oxidative DNA damage; ↑ antioxidant capacity)	↓ COX2 expression ↓ TNF-α (plasma) ↓ DNA damage ↑ plasma total antioxidant power Prevention of all LPS-induced inflammatory effects	[200]
Hyperlipidemic rabbits	different diets: control, atherogenic, EVOO, or HTyr-supplemented (4 mg/kg)	HTyr: ↑ antioxidant status ↓ atherosclerotic lesion progression (reduced intimal layer area) Modifying blood lipids	Atherogenic diet for 1 month followed by 1 month of HT + control diet: ↓ TC (50%) ↓ TG (42%) ↑ HDL (2.3-fold) Atherogenic diet + HTyr for 1 month: ↓ intimal layer area of aortic arch ↑ TAC ↓ MDA	[208]
Young Wistar rats (with diet-induced metabolic syndrome)	HTyr (with or without 20 mg/kg/day, oral gavage), 8 weeks, high-carbohydrate high-fat diet for both groups	↑ lipid mobilization ↑ branched fatty acid esters of hydroxy-oleic acids (OAHSAs) ↓ hepatic steatosis ↓ hepatic inflammation	↓ hepatic steatosis ↓ inflammatory cell infiltration in liver ↑ OAHSA → improving metabolic syndrome	[213]
Mice with diet-induced obesity and Human heptoma cell line HepG2	HTyr (20 mg/kg/day, then for cells: 100 μM), 10 weeks, (3 groups: control, high-fat diet, high-fat diet + HTyr)	Modulating endoplasmic reticulum (ER) stress, insulin signaling Anti-inflammatory effects Regulating lipid metabolism	↓ FBG, ↓ fasting insulin ↑ HOMA-IR ↓ hs-CRP, TNF-α, IL-6, IL-1β ↔ TC, ↔ LDL, ↑ HDL, ↔ TG, ↔ BW ↓ hepatic steatosis ↓ key ER stress sensors: p-PERK, p-IRE1α, ATF6, GRP78 (in both adipose tissue and liver) JNK/IRS/Akt Insulin Signaling Pathway: ↓ p-JNK, ↑ IRS-1 Tyr phosphorylation, ↑ Akt Ser473 phosphorylation (Restores insulin signaling in both liver and in HepG2 cells) ↓ SREBP1 activation ↓ mRNA expression of downstream lipogenic enzymes: ACC1, FAS, SCD1 ↓ ER swelling and dilation in hepatocytes caused by high-fat diet	[214]
C57BL/6J male mice fed a high-fat diet (HFD) vs. control diet	HTyr (5 mg/kg/day), 12 weeks Groups: Control, Control + HTyr, HFD, HFD + HTyr	Improving PUFA profile ↓ lipogenesis ↑ antioxidant capacity ↓ inflammation	Compared to Control: ↑ DHA (C22:6 n-3) ↑ Total n-3 LCPUFA ↑ Total LCPUFA ↓ n-6/n-3 ratio ↑ Nrf2-related antioxidant enzymes (Nrf2 mRNA, ↑ Nrf2 DNA binding, ↑ GST mRNA, ↑ GCL mRNA) Compared to HFD: ↓ TBARS, F-2 isoprostanes, protein carbonyls ↑ CAT activity, SOD activity, GPx activity, GR activity, Nrf2 DNA-binding activity ↑ Nrf2 mRNA, GCL mRNA, GST mRNA ↓ white adipose tissue and TAG content per adipocyte ↑ adipocyte number (per gram tissue) ↑ Adiponectin ↓ Leptin ↓ SREBP-1c pathway (**↓** SREBP-1c DNA-binding, SREBP-1c mRNA, FAS mRNA & activity, ACC mRNA and activity, G6PD activity, Malic enzyme activity) ↓ NF-κB pathway (↓ NF-κB DNA-binding, ↓ NF-κB mRNA, ↓ TNF-α mRNA, ↓ IL-6 mRNA)	[215]
Streptozotocin (STZ)-induced diabetic rats	Tyr (20 mg/kg), oral, for 45 days, 5 groups	Glycemic regulation ↑ antioxidant Defense ↓ inflammation ↓ lipid peroxidation	↓ glucose, ↑ insulin ↑ SOD, CAT, GPx, GSH, GR ↓ CRP, NF-κB p65, TNF-α, ↓ IL-6 ↓ TBARS, LOOH	[218]
In vitro				
Human endothelial cells (EA.hy926)	HTyr (10 nM–100 µM), 15 min-24 h	eNOS pathway (promoter activity, enzyme activity, NO availability)	↔ No significant effect on eNOS activity, eNOS promoter activation, or NO availability	[167]
ECV304 endothelial cells (T2D-like model: high glucose + FFA)	HTyr and EVOO polyphenol extract	↓ ROS ↑ eNOS phosphorylation ↑ NO ↓ ET-1; modulation of intracellular Ca^2+^	↑ NO, ↓ ET-1, ↓ ROS, ↑ eNOS-P	[168]
HAECs co-incubated with TNF-α; HTyr metabolites produced using Caco-2 cells	HTyr and HTyr metabolites (1–10 μM, 18–24 h)	↓ endothelial adhesion molecules ↓ inflammatory chemokine secretion	↓ E-selectin, ↓ P-selectin, ↓ ICAM-1, ↓ VCAM-1; HTyr metabolites also ↓ MCP-1 (24 h)	[169]
THP-1-derived macrophages	EVOO polyphenol concentrate (EVOO-PC; up to 320 µg/mL); EVOO-PC-enriched HDL	↑ ABCA1-mediated cholesterol efflux ↑ protection of HDL efflux under oxidative stress	↑ cholesterol efflux (~40% ↑) ↑ efflux preserved under oxidative conditions	[170]
J774 macrophages	HTyr (0–25 µM) Tyr (0–25 µM) EVOO-PC (0–320 µg/mL)	↑ ABCA1 expression ↑ ABCA1-dependent cholesterol efflux	↑ cholesterol efflux (dose-dependent) ↑ ABCA1 protein	[170]
Endothelial cells challenged with PMA (inflammatory endothelial dysfunction model)	HTyr 1–30 µM, pretreatment	↓ mitochondrial superoxide ↓ lipid peroxidation ↑ SOD activity ↑ mitochondrial membrane potential ↑ ATP synthesis ↑ ATP5β ↑ PGC-1α, NRF-1, TFAM (mitochondrial biogenesis)	↓ mtROS ↓ lipid peroxidation ↑ SOD ↑ ATP ↑ ATP5β ↑ mtDNA ↑ PGC-1α ↑ NRF-1 ↑ TFAM ↓ pathological angiogenesis	[182]
H9c2 cardiomyocytes exposed to X/XO (oxidative toxicity model)	HTyr 0.1–10 µg/mL, 24 h pretreatment	↓ ROS ↓ MAPKAPK-2-P ↓ cleaved caspase-3 ↑ c-Jun-P ↑ p44/42-MAPK-P ↑ Hsp27-P	↑ cell viability ↓ ROS ↓ MAPKAPK-2 phosphorylation ↓ cleaved caspase-3 ↑ c-Jun phosphorylation ↑ p44/42-MAPK phosphorylation ↑ Hsp27 phosphorylation	[183]
Porcine pulmonary artery endothelial cells (VECs) exposed to H_2_O_2_	HTyr (10, 30, 50 μM); pretreatment after 12 h serum starvation	↑ AMPK phosphorylation → ↑ FOXO3a (cytosolic and nuclear) → ↑ catalase (mRNA, protein, activity) ↓ ROS	↓ ROS ↑ CAT mRNA ↑ CAT protein ↑ CAT activity ↑ AMPK-P ↑ FOXO3a ↑ FOXO3a nuclear translocation	[184]
Porcine vascular endothelial cells (VECs)	HTyr 10–100 μM, 1–24 h (dose- and time-dependent)	↑ PI3K/Akt and ↑ ERK1/2 → ↑ Nrf2 stabilization and nuclear translocation → ↑ HO-1 → enhanced endothelial repair	↑ HO-1 mRNA and protein ↑ Nrf2 nuclear accumulation ↑ Nrf2 stabilization (↑ half-life) ↑ endothelial wound healing	[187]
J774A.1 macrophages (LDL oxidation model)	HTyr/Tyr (1.5 µM-0.5 mM) (exposures 2–24 h)	Antioxidant defense preservation (↑ GSH system; ↓ intracellular ROS) Inhibition of LDL oxidation Intracellular accumulation dynamics differing between HTyr and Tyr	↓ cell-mediated LDL oxidation (HTyr ~ 100% inhibition; Tyr ~ 40%) ↓ ROS (HTyr early; Tyr at 12–24 h) ↑ GSH ↑ GSH-related enzyme activities Prevention of antioxidant defense impairment HTyr rapidly taken up then cleared Tyr accumulates over time	[193]
L6 skeletal muscle cells under H_2_O_2_-induced oxidative stress	Tyr at 1, 30, 100 μM, Co-treated with 0.5 mM H_2_O_2_, incubated for 24 h	Regulation of ERK, JNK, p38 MAPK ↑ HO-1	↑ Cell viability ↓ cleaved caspase-3 ↓ p-ERK ↓ p-p38 ↓ p-JNK ↑ ATP ↑ HO-1	[194]
Rat peritoneal mast cells isolated from adult male Wistar rats	HTyr and oleuropein (Preincubation with 10, 50, 100, 200, 400 μM for 5, 10, 20, or 45 min at 37 °C)	Mast cell stabilization Inhibition of β-hexosaminidase release (marker of degranulation)	↓ Mast cell degranulation (dose-dependent) under all stimuli (Con A, Cpd 48/80, A23187) HTyr > sodium cromoglycate at 100 μM (Con A challenge) Oleuropein > sodium cromoglycate at 10–100 μM (A23187 challenge) No reduction in cell viability	[204]
THP-1 macrophage-derived foam cells (PMA-differentiated 48 h; ox-LDL 50 µg/mL for 24 h to induce foam cells)	HTyr 50 µM for 24 h (after foam-cell induction). Viability tested at 25–2000 µM. Inhibitors: GW9662 10 µM (PPARγ antagonist) and GSK2033 1 µM (LXRα inhibitor) added 30 min before HTyr	Activation of PPARγ → LXRα → ABCA1 pathway → (reducing cholesterol accumulation in foam cells) [205] ↑ ABCA1, ↑ ABCG1 ↓ SR-A1, ↓ CD36, ↓ LOX-1 (cholesterol metabolism-related molecules) ↓ adhesion factors ↓ pro-inflammatory factors	↓ Total cholesterol (TC) ↓ Free cholesterol (FC) ↓ Foam-cell formation (Oil Red O) ↑ Cholesterol efflux ↓ THP-1 adhesion to LPS-stimulated HUVECs ↓ ICAM-1, ↓ VCAM-1 ↓ TNF-α, ↓ IL-6 (NF-κB-related cytokines)	[205]
RAW264.7 murine macrophages (LPS-induced inflammatory model)	Tyr 1.2 mM co-treatment with LPS	Tyr inhibits LPS-induced upstream inflammatory signaling ↓ activation of TLR4-MyD88/TRIF pathways ↓ pro-inflammatory response via ↑ mCD14 cleavage and ↓ receptor availability for LPS	↓ LPS-macrophage binding (CD14-mediated) ↓ Membrane-bound CD14 (mCD14) expression ↓ TLR4/MD2 complex expression ↓ MyD88 concentration ↓ TRIF concentration ↑ Soluble CD14	[206]
Primary human visceral adipocytes	HTyr (5, 10, 30, and 70 µg/mL), from the first day of differentiation till 7 days post differentiation	Regulating adipogenesis and lipid metabolism by: ↑ expression of genes that inhibit adipogenesis ↓ expression of genes that promote adipogenesis	↑ Lipolysis ↑ Apoptosis ↓ TG accumulation ↑ GATA2, ↑ GATA3, ↑ WNT3A, ↑ SFRP5, ↑ HES1, ↑ SIRT1 (Anti-adipogenic gene) ↓ LEP, ↓ FGF1, ↓ CCND1, ↓ SREBF1 (Pro-adipogenic gene) (Effects were seen only during differentiation, not in mature adipocytes (A7))	[212]

Note: Arrows indicate the direction of significant changes observed in the studies: ↓ denotes a significant decrease, ↑ denotes a significant increase, and ↔ indicates no significant change. Abbreviations: ABCA1, ATP-binding cassette transporter A1; ABCG1, ATP-binding cassette transporter G1; ACC, acetyl-CoA carboxylase; AMPK, AMP-activated protein kinase; ApoAI, apolipoprotein A-I; ATF6, activating transcription factor-6; ATP5β, ATP synthase beta subunit; CAT, catalase; COX-2, cyclooxygenase-2; CRP, C-reactive protein; eNOS, endothelial nitric oxide synthase; ER, endoplasmic reticulum; ET-1, endothelin-1; FAS, fatty acid synthase; FBG, fasting blood glucose; FOXO3a, forkhead box O3a; FFA, free fatty acids; GCL, glutamate cysteine ligase; GPx, glutathione peroxidase; GR, glutathione reductase; GRP78, glucose-regulated protein 78; GSH, reduced glutathione; GST, glutathione S-transferase; HAECs, human aortic endothelial cells; HDL, high-density lipoprotein; HMGR, HMG-CoA reductase; HO-1, heme oxygenase-1; HOMA-IR, homeostatic model assessment of insulin resistance; Hsp27, heat shock protein-27; ICAM-1, intercellular adhesion molecule-1; IL-1β, interleukin-1β; IL-6, interleukin-6; IL-10, interleukin-10; IRE1α, inositol-requiring enzyme-1 alpha; IRS-1, insulin receptor substrate-1; JNK, c-Jun N-terminal kinase; LDL, low-density lipoprotein; LDLR, low-density lipoprotein receptor; LOOH: lipid hydroperoxide, LPS, lipopolysaccharide; LXRα, liver X receptor-alpha; MAPK, mitogen-activated protein kinase; MAPKAPK-2, MAPK-activated protein kinase-2; MCP-1, monocyte chemoattractant protein-1; MDA, malondialdehyde; MI, myocardial infarction; mtROS, mitochondrial reactive oxygen species; NF-κB, nuclear factor kappa-B; NO, nitric oxide; NRF-1, nuclear respiratory factor-1; Nrf2, nuclear factor erythroid 2–related factor-2; OAHSA: Oxidized And Hydroxylated Synthetic Acids, PCSK9, proprotein convertase subtilisin/kexin type-9; PERK, protein kinase RNA-like ER kinase; PGC-1α, PPARγ, peroxisome proliferator-activated receptor gamma; PUFA, polyunsaturated fatty acids; PWV, pulse wave velocity; ROS, reactive oxygen species; SCD1, stearoyl-CoA desaturase-1; SIRT1, sirtuin-1; SOD, superoxide dismutase; SR-A1, scavenger receptor class A1; SREBP-1c, sterol regulatory element-binding protein-1c; STZ, streptozotocin; T2D, type 2 diabetes; TAC, total antioxidant capacity; TBARS, thiobarbituric acid reactive substances; TFAM, mitochondrial transcription factor-A; TLR4, Toll-like receptor-4; TNF-α, tumor necrosis factor-α; VCAM-1, vascular cell adhesion molecule-1.

## Data Availability

The original contributions presented in the study are included in the article, further inquiries can be directed to the corresponding author.

## Abbreviations

The following abbreviations are used in this manuscript:

ABCA1	ATP binding cassette subfamily A member 1
ABCG1	ATP binding cassette subfamily G member 1
ACC	Acetyl-CoA carboxylase 1
ADH	Alcohol dehydrogenase
ALDH	Aldehyde dehydrogenase
ALR	Aldehyde reductase
AMPK	Activated AMP-activated protein kinase
APC	Antigen presenting cell
Apo	Apolipoprotein
ASCVD	Atherosclerotic cardiovascular disease
ATP	Adenosine triphosphate
CA	Cholic acid
CAT	Catalase
Ca^2+^	Calcium ion
CaMKII	Calmodulin-dependent protein kinase II
CD36	Cluster of differentiation 36
CDCA	Chenodeoxycholic acid
CFR	Coronary flow reserve
COMT	Catechol-O-methyltransferase
COX	Cyclooxygenase
CRP	C-reactive protein
CVD	Cardiovascular disease
CYP	Cytochrome P
DC	Dendritic cell
DCA	Deoxycholic acid
DM	Diabetes mellitus
DNA	Deoxyribonucleic acid
DOPAC	3,4-dihydroxyphenylacetic acid
EA	Ellagic acid
EFSA	European Food Safety Authority
eNOS	Endothelial nitric oxide synthase
ERK	Extracellular signal-regulated kinase
ET-1	Endothelin-1
FAS	Fatty acid synthase
FFAR2	Free fatty acid receptor 2
FMD	Flow-mediated dilation
FMO3	Flavin-containing monooxygenase 3
FXR	Farnesoid X receptor
GALT	Gut-associated lymphoid tissue
GLP-1	Glucagon-like peptide-1
GPR109A	G protein-coupled receptor 109A
GPx	Glutathione peroxidase
HDAC	Histone deacetylase
HDL	High-density lipoprotein
HMGR	3-hydroxy-3-methyl-glutaryl-CoA reductase
4-HPAA	4-hydroxyphenylacetic acid
4-HPAL	4-hydroxyphenylacetaldehyde
hs-CRP	High-sensitivity C-reactive protein
HTyr	Hydroxytyrosol
HVA	Homovanillic acid
ICAM-1	Intercellular adhesion molecule 1
IFNγ	Interferon gamma
IgA	Immunoglobulin A
IL	Interleukin
ITS	Internal transcribed spacer
JNK	Jun N-terminal kinase
LCA	Lithocholic acid
LDH	Lactate dehydrogenase
LDL	Low-density lipoprotein
LOX	Lipoxygenase
LPS	Lipopolysaccharides
LXRα	Liver X Receptor Alpha
MAIT	Mucosal-associated invariant T
MAO	Monoamine oxidase
MAPK	Mitogen-activated protein kinase
MCP-1	Monocyte chemoattractant protein 1
MerTK	Mer proto-oncogene tyrosine kinase
NF-κB	Nuclear factor kappa B
NKT	Natural killer T
NO	Nitric oxide
NOS	Nitric oxide synthase
Npc1l1	Niemann-Pick C1-like1
NRF-1	Nuclear respiratory factor-1
Nrf2	Nuclear factor-E2-related factor 2
Ox-LDL	Oxidized low-density lipoprotein
PA	Propionic acid
PAGln	Phenylacetylglutamine
PBR	Perfused boundary region
PCSK9	Proprotein Convertase Subtilisin/Kexin Type 9
PDE	Phosphodiesterase
PERK	Protein kinase R-like endoplasmic reticulum kinase
PGC-1α	Peroxisome proliferator-activated receptor gamma coactivator-1α
PLC β3	Phospholipase C β3
PPyA	Phenylpyruvic acid
PPARγ	Peroxisome Proliferator-Activated Receptor Gamma
PSA	Polysaccharide A
PWV	Pulse wave velocity
RCT	Reverse cholesterol transport
rRNA	ribosomal Ribonucleic acid
ROS	Reactive oxygen species
SCD1	Stearoyl-CoA desaturase 1
SCFA	Short-chain fatty acid
SOD	Superoxide dismutase
SR-A1	Scavenger receptor class A1
SREBP	Sterol regulatory element-binding protein
SULT	Sulfotransferase
TDC	Tyrosine decarboxylase
T2DM	Type 2 diabetes mellitus
TF	Tissue factor
TFAM	Mitochondrial transcription factor A
TG	Triglyceride
TGF-β	Transforming growth factor beta
TGR5	Takeda G protein-coupled receptor 5
Th	T-helper
TJP	Tight junction proteins
TLR2	Toll-like receptor 2
TMA	Trimethylamine
TMAO	Trimethylamine N-oxide
TNF-α	Tumor necrosis factor alpha
Tregs	Regulatory T cells
Tyr	Tyrosol
UGT	UDP-glucuronosyltransferase
VCAM1	Vascular cell adhesion molecule 1
VEGF	Vascular endothelial growth factor
VLDL	Very- low-density lipoprotein
WAT	White adipose tissue
